# Tracing the evolution of nectin and nectin-like cell adhesion molecules

**DOI:** 10.1038/s41598-020-66461-4

**Published:** 2020-06-10

**Authors:** Kheerthana Duraivelan, Dibyendu Samanta

**Affiliations:** School of Bioscience, Indian Institute of Technology Kharagpur, Kharagpur, 721302 West Bengal India

**Keywords:** Biochemistry, Cell biology, Computational biology and bioinformatics, Genetics

## Abstract

Nectin and nectin-like cell adhesion molecules (collectively referred as nectin family henceforth) are known to mediate cell-cell adhesion and related functions. While current literature suggests that nectins are prevalent in vertebrates, there are no in-depth analyses regarding the evolution of nectin family as a whole. In this work, we examine the evolutionary origin of the nectin family, using selected multicellular metazoans representing diverse clades whose whole genome sequencing data is available. Our results show that this family may have appeared earlier during metazoan evolution than previously believed. Systematic analyses indicate the order in which various members of nectin family seem to have evolved, with some nectin-like molecules appearing first, followed by the evolution of other members. Furthermore, we also found a few possible ancient homologues of nectins. While our study confirms the previous grouping of the nectin family into nectins and nectin-like molecules, it also shows poliovirus receptor (PVR/nectin-like-5) to possess characteristics that are intermediate between these two groups. Interestingly, except for PVR, the other nectins show surprising sequence conservations across species, suggesting evolutionary constraints due to critical roles played by these proteins.

## Introduction

Cell-cell adhesion is one of the most fundamental processes, indispensable for the development and maintenance of multicellular organisms^1–3^. A diverse group of cell surface glycoproteins, collectively called cell adhesion molecules (CAMs) play a major role at the adhesion sites. These CAMs are mainly members of 4 families in higher metazoans: selectins, cadherins, integrins and immunoglobulin superfamily (IgSF) proteins^4^. The nectin family, consisting of 9 homologues in humans, belongs to the IgSF. Nectin family consists of both nectins (nectin-1 to -4) and nectin-like molecules (nectin-like-1 to -5). They are membrane-bound, single-pass, type-I glycoproteins containing multiple immunoglobulin (Ig) folds in their extracellular domains and found to be involved in the formation of cell-cell junctions, especially adherens junctions^4,5^. Initially, each member of the nectin family was independently identified by multiple research groups and named based on the observed functions, due to which they possess multiple names (Table 1)^3,6^.

**Table 1:** List of alternate names of nectin family members.

Protein	Alternate names	Gene names
Necl-1	CADM3, IgSF4B, SynCAM3, TSLL1, Brain Ig receptor	*NECL1, CADM3, IGSF4B, SYNCAM3, TSLL1*
Necl-2	CADM1, IgSF4, SynCAM, TSLC1, SgIgSF	*NECL2, CADM1, IGSF4, IGSF4A, SYNCAM, TSLC1*
Necl-3	CADM2, IgSF4D, SynCAM2	*NECL3, CADM2, IGSF4D*
Necl-4	CADM4, IgSF4C, TSLL2	*NECL4, CADM4, IGSF4C, TSLL2*
Necl-5	CD155, PVR	*PVR, PVS*
Nectin-1	CD111, PVRL-1, PRR-1, HIgR, HveC	*NECTIN1, HVEC, PRR1, PVRL1*
Nectin-2	CD112, PVRL-2, PRR-2, HveB	*NECTIN2, HVEB, PRR2, PVRL2*
Nectin-3	CD113, PVRL-3, PRR-3	*NECTIN3, PRR3, PVRL3*
Nectin-4	PVRL-4, PRR-4, IgSF receptor LNIR, EDSS1	*NECTIN4, LNIR, PRR4, PVRL4*

Abbreviations: CADM-Cell adhesion molecule; IgSF-Immunoglobulin superfamily; SynCAM-Synaptic cell adhesion molecule; PVR-Poliovirus receptor; Necl-Nectin-like; Hve-Herpes virus entry mediator; HIgR-Herpesvirus Ig-like receptor; CD-Cluster of differentiation; TSLC-Tumour suppressor in lung cancer; TSLL-Tumour suppressor in lung cancer like; SgIgSF-Spermatogenic immunoglobulin superfamily; PVRL-Poliovirus receptor-like; PRR-Poliovirus receptor-related; EDSS-Ectodermal dysplasia-syndactyly syndrome. All names and abbreviations sourced from www.uniprot.org.

Although nectins were initially discovered as viral entry receptors, their involvement in cell-cell adhesion and immune modulation has gained importance in recent years^4,7^. This adhesion function is attributed to the ability of these proteins to interact with a number of cell surface proteins through their ectodomains. Moreover, cytoplasmic tails of these proteins also bind to the actin cytoskeleton through adaptor molecules like afadin, band 4.1 protein, etc., and thereby physically connect adjacent cells^2,8,9^. The nectin family is diverse in terms of their tissue specificity and functions^3,10–17^. Nectin-mediated cell-cell adhesions regulate a wide variety of functions including epithelial adherens junction formation, neurulation, spermatogenesis, brain development, etc.^3,5,8,18,19^.

While structure-function relations of the nectin family members have been extensively explored in recent years^7,20,21^, systematic studies regarding the evolutionary origin of the nectin family are yet to be carried out. Although current literature suggests that nectins are prevalent in vertebrates, with orthologues in *Drosophila melanogaster*, these assertions are not based upon any systematic studies^22^. In this work, we have examined the evolutionary origin of the 9 human nectin homologues, using selected multicellular metazoans. Since nectins represent a diverse family, we first defined the exact properties of each of the 9 human nectin homologues. Standard protein BLAST search against selected metazoans was carried out with the 9 human nectin proteins as the query, and the hits from each organism were classified based on our definition of nectins. Using these data, we have mapped the appearance of each nectin family protein on a phylogenetic tree of metazoan life. During this study, we also identified the presence of clade-specific proteins resembling nectins, but with no obvious orthologues in humans. Furthermore, the current study also reveals the presence of possible ancient homologues of the nectin family in some of the primitive clades.

## Results

### The 9 human nectin homologues cluster into 2 distinct sub-groups

**Figure 1:**
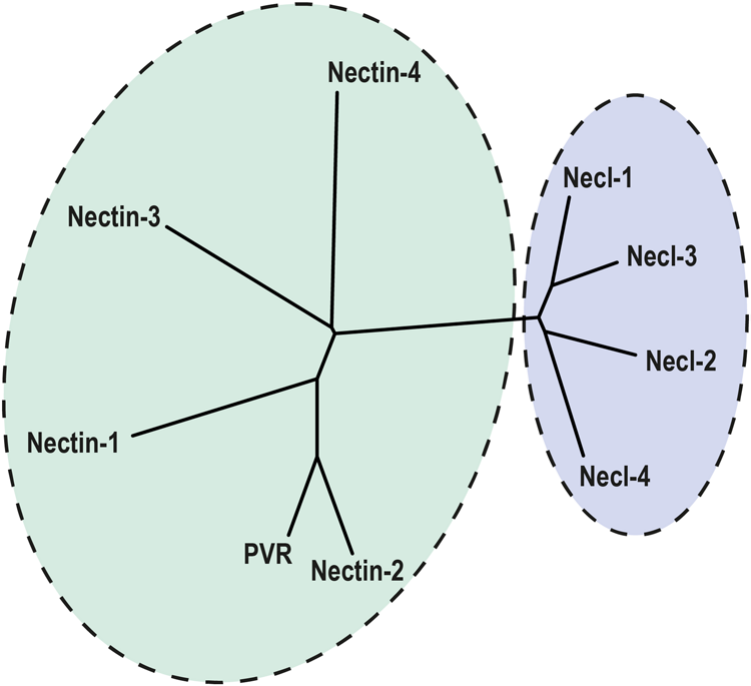
Phylogenetic tree of the 9 human nectin homologues. The evolutionary relationship between the 9 human nectin homologues was analysed with an unrooted phylogenetic tree created with PHYLIP package, using full-length sequences. The results indicate that the nectin family cluster into 2 distinct sub-groups, one consisting of 5 members: nectin-1 to -4 and PVR; the other with necl-1 to -4.

The immunoglobulin superfamily consists of proteins with varying characteristics, with the only shared feature being the presence of at least one Ig-like fold^23,24^. Since the presence of an Ig fold alone cannot be used as a predictor for a protein to belong to the nectin family, there arises a need to define and characterize each of the nectins based on overall sequence profiles, motifs and patterns, apart from analysing the domain organizations. In order to do this, we first aligned the full-length sequences of all 9 human nectins (Supplementary Fig. S1). The results show some conserved residues in the entire family, but no exact regions of conservation. This was expected, since nectin family consist of two different sub-families: nectins and the nectin-like molecules. In order to exactly find out which members of the nectin family cluster together, we constructed a phylogenetic tree of the 9 human nectins using PHYLIP package (Fig. 1). The phylogenetic tree shows that nectin-like-1, -2, -3 and -4 cluster together into one sub-group (necls), while all 4 nectins and nectin-like-5 (also known as PVR) form a second sub-group (nectins).

### Each of the 9 human nectin homologues show distinct sequence conservation patterns

**Figure 2:**
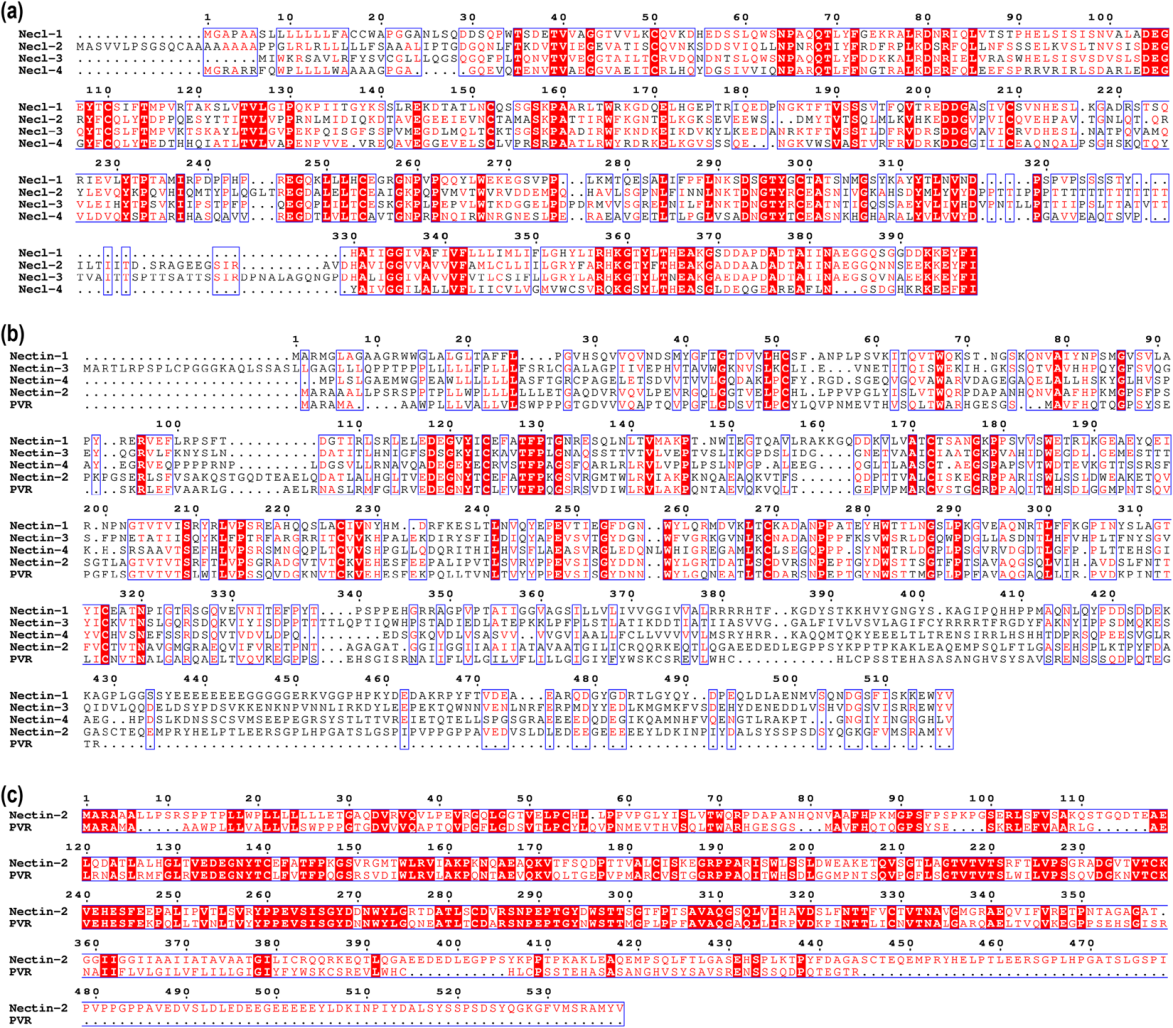
Multiple sequence alignments of the 2 sub-groups of the nectin family. Nectin-like molecules and nectins were aligned to look for consensus sequences. Nectin-like molecules **(a)** show higher sequence similarity compared to nectins **(b)**. In nectins, the cytoplasmic region is not conserved, while in necls, even the cytoplasmic region shows high sequence similarities. Nectin-2 and PVR show high sequence identity **(c)**, suggesting that they share a common ancestry.

Since the family consists of 2 sub-groups, it is clear that the 2 sub-groups must be dealt with separately while characterizing the proteins. Accordingly, multiple sequence alignments were performed with the 2 sub-groups (Fig. 2a,b), which show key conservation regions of the 2 sub-group members. Of special interest is the alignment between nectin-2 and PVR (Fig. 2c), discussed in detail later.

**Figure 3:**
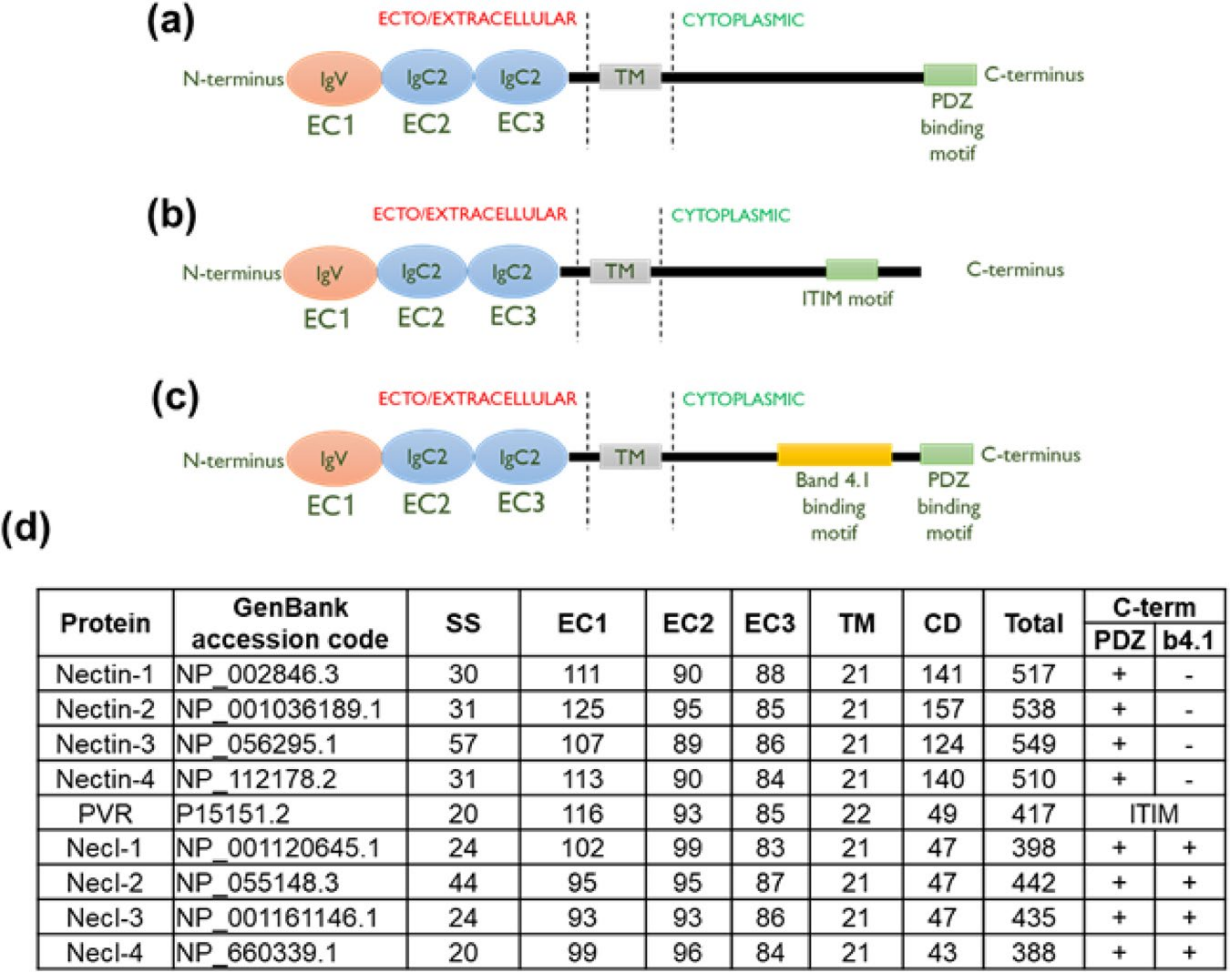
Domain organization of nectin family members. Based on the multiple sequence alignments performed earlier, the domain organization of nectins are represented here. All 9 nectins have an extracellular region (EC) composed of 3 domains – an Ig V-like domain (EC1) followed by 2 Ig-C-like domains (EC2 and EC3), a transmembrane region (TM) and a cytoplasmic domain (CD), in addition to a signal sequence (SS) **(a–d)**. However, there are some differences in the cytoplasmic motifs between nectins **(a)**, PVR **(b)** and necls **(c)**. Nectins **(a)** possess a PDZ-binding motif in their cytoplasm, while nectin-like molecules **(c)** contain a PDZ-binding motif as well as a band 4.1 binding motif. PVR, on the other hand contains only an ITIM motif **(b)**.

In order to define and identify the sequence characteristics of each of the 9 human nectin homologues, the known orthologues of nectins from different vertebrates (wherever possible, because some nectins are absent in some of the earlier vertebrates) were analysed for conserved sequence motifs, characteristics, and domains (Supplementary Fig. S2). The 4 human nectins show sequence similarities in their extracellular domains, but vary considerably in their cytoplasmic region, except for the presence of PDZ binding motifs in their carboxy-termini (Fig. 3a,d, Supplementary Fig. S2). However, all 4 necls show higher sequence similarities in their extracellular as well as cytoplasmic domains, and are characterized by the occurrence of a band 4.1 binding motif and a C-terminal PDZ binding motif (Fig. 3c,d, Supplementary Fig. S2). PVR however lacks both PDZ binding motif as well as band 4.1 binding motif, but instead has an immunoreceptor tyrosine-based inhibition motif (ITIM) (Fig. 3b,d).

### The evolution of the nectin family in metazoans

**Figure 4:**
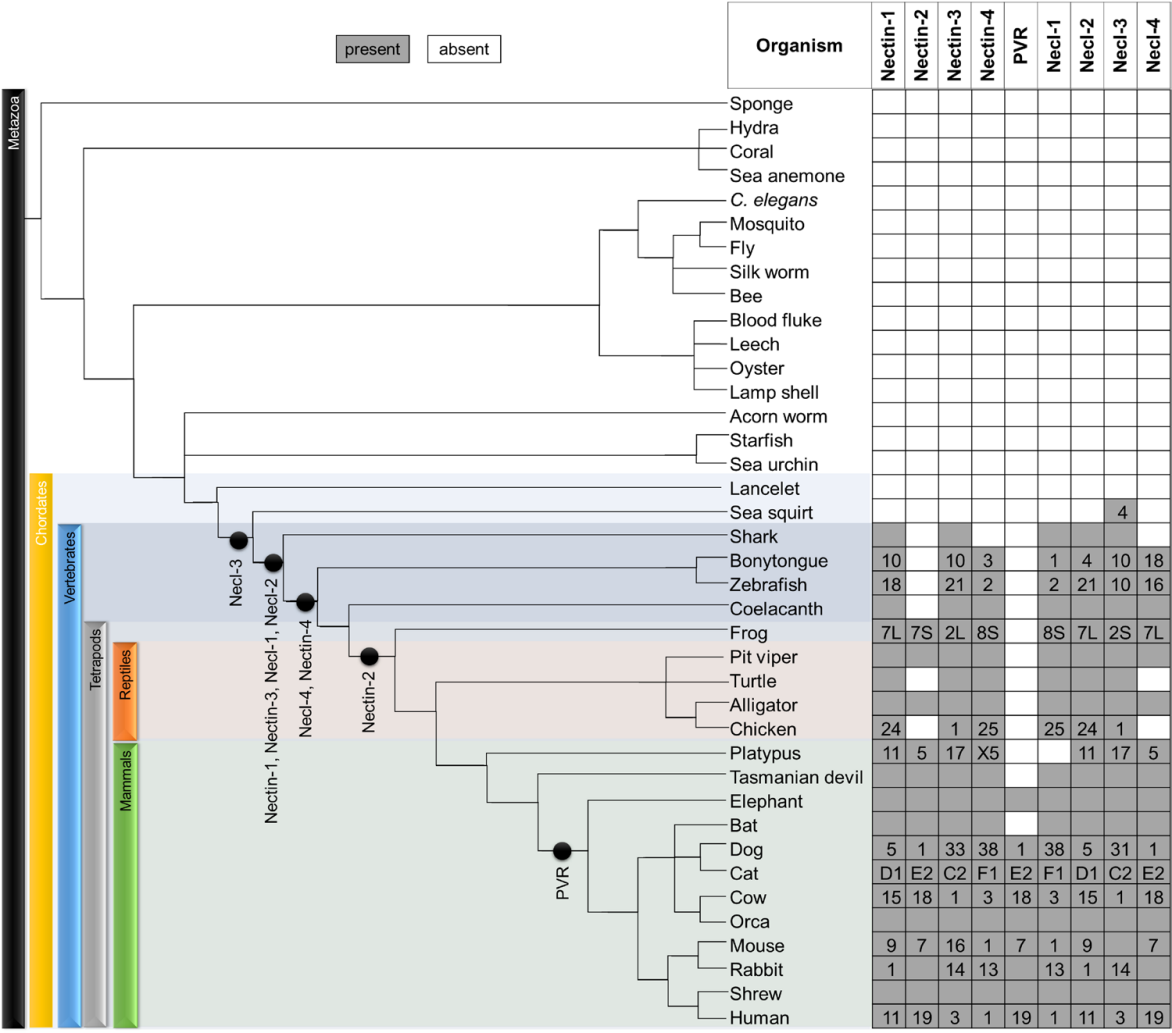
Evolution of the nectin family in metazoans. Whole genome sequences of 39 organisms belonging to diverse clades of metazoans, were used in BLASTp search against the 9 human nectin homologues. After analyses, the evolutionary appearance of nectins were plotted into the phylogenetic tree of metazoans (not to scale). The clade-specific absences of nectins are shown in the figure represented as blank cells. Also, the numbers indicate the chromosomal location of each nectin gene, which suggests that the nectin family genes show chromosomal clustering during metazoan evolution.

In order to find out at which point during metazoan evolution each of the 9 human nectin homologues appeared, 39 organisms representing diverse clades of metazoans whose whole genome sequences are currently available were chosen (Supplementary Table S1). However, the main problem here lies in the fact that protein names are just identifiers used to identify proteins. Some difficulty in directly searching for nectins in any database are that nectins have several names (Table 1), and also because nectins in several organisms still remain uncharacterized. Furthermore, unrelated proteins may have same names, for example a protein called nectin in *Paracentrotus lividus* (a purple sea urchin) with unrelated domain organizations and functions. Hence, we used blastp to find orthologues of all 9 human nectins. A database of the top blastp hit for each of the 9 proteins in all 39 organisms was built (Supplementary Table S2). Individual members from the hits database were examined for the occurrence of the characteristics described in Fig. 3, in order to decide whether the protein can be categorized into a nectin and into which nectin. Figure 4 shows the evolutionary appearance of nectins on a phylogenetic tree consisting of 39 representative metazoans. It was observed that necl-3 appeared first in early chordates (sea squirt), followed by appearance of nectin-1 and -3, necl-1, and -2 in early vertebrates (shark). The most recently evolved member is PVR, which is present only in placental mammals (Fig. 4).

### Taxon-specific absences of some nectins

During our study, we found that some clades seem to have lost nectin-2 and PVR, while it is present in other organisms, diverged earlier as well as other clades under the same higher classification (Fig. 4). For e.g., PVR is present in placental mammals, but bats of the Vespertilionidae family lack this protein. Similarly, nectin-2 evolved in tetrapods, but is absent in turtles and most birds (nectin-2 is present only in some flightless birds like emu).

### Presence of proteins related to nectins, but not present in humans

**Figure 5:**
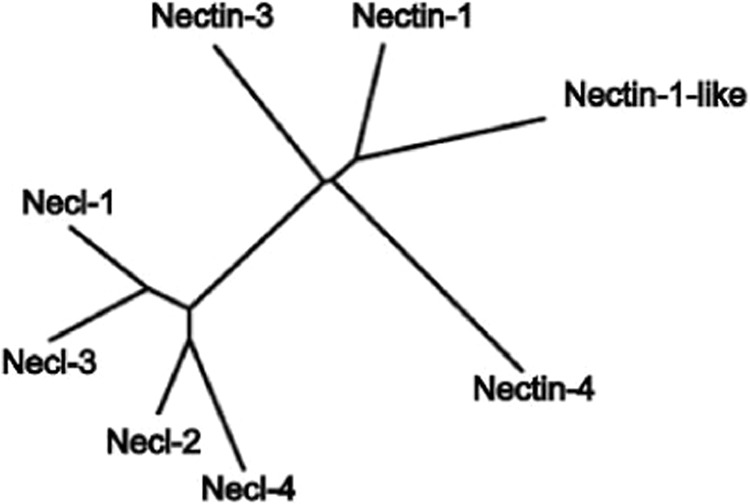
Proteins with equidistant relationship to more than one human nectin. Coelacanth, a primitive fish contains nectin-1 and nectin-1-like, and lacks nectin-2. Since reverse blast to human proteome shows that nectin-1-like is equally related to both human nectin-1 and nectin-2, nectin-1-like may represent an intermediate between these two proteins.

During the course of this study, some taxon-specific proteins related to human nectin family members but not present in humans were found. During reverse-BLAST, we noticed that these proteins give somewhat equidistant scores/e-values with more than one human nectins. Especially, snakes and frogs possess more than one such protein. The nectin-1-like (not nectin-like-1) in coelacanth is intermediate to human nectin-1 and nectin-2 (Fig. 5). We hypothesize that these extra nectins may in fact represent transition state proteins, wherein one nectin diverged from another.

### Presence of possible nectin homologues in evolutionarily primitive organisms

As mentioned, nectins are present only in vertebrates, while necl-3 can be traced from early chordates (Fig. 4). During our study, we came across a few hits that do not resemble nectins as such, but possess domain organizations similar to nectins and some additional unique domains (Table 2). These proteins are unique to the respective phyla (based on thorough BLAST studies) -for e.g., the protein from Florida lancelet seems to be present only in the genus *Branchiostoma*. Also, the domain organization of the protein is not found elsewhere (Table 2). This led us to hypothesize that these proteins may be ancient homologues, having arisen from similar proteins as nectins, but underwent a different path during subsequent evolution to form proteins with unique domain organizations.

**Table 2:** Possible ancient homologues of the nectin family.

Organism	Domains	Description
Florida lancelet	IgV/IgC/IgC/TM/SH3	Src homology 3 domains containing proteins are involved in signal transduction and connecting membrane proteins to the cytoskeleton
Starfish	TM/(Ig)9/FN3/TM/snRNP	small nuclear ribonucleoprotein particles -involved in pre-mRNA splicing
Starfish	SRCR/(Ig)4/TM	scavenger receptor cysteine-rich (SRCR) - involved in specific ligand binding in immune system
Sea squirt	Ig/IgC/IgC/TM/dnaA superfamily	DnaA superfamily- initiation of bacterial chromosomal replication

## Discussion

Currently, no studies are available that systematically explore the evolutionary origin of nectin family members that includes both nectin and nectin-like cell adhesion molecules. In this study, we have traced the evolution of the nectin family of proteins which provides further insights into the cell adhesion molecules in metazoans. The current study successfully revealed that this family of proteins may have appeared earlier during metazoan evolution than previously believed and also the order in which various members of nectin family may have evolved.

Despite being diverse in terms of function as well as phylogenetic distribution, members of the IgSF share a common structural scaffold called “Ig-fold”^23,24^. Hence, the presence of an Ig-like fold alone cannot be used as a criterion while searching for nectin homologues. Thus, a number of additional parameters such as sequence pattern and conservation were taken into consideration, in addition to the presence of Ig-fold, while searching for nectin homologues. The human nectin family consists of 9 members that are clustered into 2 sub-groups: nectins and nectin-like molecules (Fig. 1). Although individual members of the nectin family show distinct conservation patterns across species, this became apparent only after multiple sequence alignment (Supplementary Fig. S2). While nectins (nectin-1 to -4) and nectin-like molecules (necl-1 to -4) contain a PDZ binding motif in their cytoplasmic tail that helps to recognize actin-binding proteins like afadin, a characteristic band 4.1 binding motif is present in the cytoplasmic tail of necls only which recognize protein 4.1 (a protein that associates with several types of cytoskeletal proteins such as actin, spectrin, etc.)^25^ (Fig. 3). Moreover, PDZ binding motifs and band 4.1 binding motifs are conserved across clades as depicted in Supplementary Fig. S2. However, PVR is an exception as it lacks both PDZ- and band 4.1 binding motifs, but contains an immunoreceptor tyrosine-based inhibition motif (ITIM-a characteristic feature of immune receptors)^26^ (Fig. 3b).

Nectins and nectin-like molecules are cell surface glycoproteins, showing diversity in tissue specificity, glycosylation as well as and intron-exon structures, which all contribute to the regulation of functions exhibited by these CAMs. Extensive bioinformatics and molecular analysis of nectin-like molecules and individual nectins have shown the presence of multiple N- and O-linked glycosylation sites^6,11–17^. An analysis of predicted N- and O-glycosylation sites of the 9 human nectins shows that these modification sites are highly conserved across species (Supplementary Fig. S2). Multiple splice variants have also been reported in every nectin family member, except necl-4. It would be interesting to see the extent of functional conservation among the different splice variants across species.

The results of this study are in good agreement with the earlier consensus that nectins are prevalent in vertebrates^6,7,27^. Furthermore, the current study provides extensive analyses regarding the appearance of each of the 9 human nectin homologues during metazoan evolution (Fig. 4). It is evident from current study that necl-3 appeared earlier when compared to other nectin family members: sea squirt, a member of tunicate (which evolved earlier than vertebrate) possesses necl-3 (Fig. 4).

Of special interest is the poliovirus receptor (PVR or necl-5). PVR seems to be the most recent addition to the nectin family, being present only in placental mammals (Fig. 4). Although PVR has historically been grouped with nectin-like molecules, it is actually closer to the nectins and seems to have diverged from nectin-2 (Fig. 1). In fact, recent studies also suggest that PVR should be grouped into nectin sub-group instead of nectin-like molecules^28^. While PVR does not possess a long cytoplasmic region like the nectins (Fig. 3b,d), it does have considerable sequence identity (~50%) with nectin-2 in human (Fig. 2c). Human nectin-2 as query delivers PVR as the top hit in mouse; similarly, human PVR as query gives nectin-2 as the top hit in rabbit. Moreover, nectin-2 and PVR also share common binding partners: nectin-3, CD226 and TIGIT (CD226 and TIGIT are prominent immune receptors in mammals). Furthermore, PVR possesses low sequence conservation across species compared to the rest of the family (Supplementary Fig. S2), suggesting that PVR is rapidly evolving, a common characteristic of most of the immune receptors^29–31^, which is again corroborated by the presence of ITIM motif in the cytoplasmic region of PVR. Taken together, we conclude that PVR diverged recently from nectin-2, and also propose that PVR should not be grouped with the nectins or the nectin-like sub-groups, as it might in fact denote a new branching of the nectin family.

During this study, we found that some early vertebrates like zebrafish, frogs, alligators, snakes and birds have some proteins which are related to nectin family, but have equidistant relationship to more than one human nectin homolog. Some of them are unique nectin homologues, with no apparent counterpart in humans. As an example, the protein named nectin-1-like (not to be confused with nectin-like-1) in coelacanth, which has equidistant relationship from human nectin-1 and nectin-2, seems to be a transition state between nectin-1 and -2 (Fig. 5). An extensive analysis reveals the presence of several such proteins in some specific clades, especially in Actinopterygii (bony fishes), Amphibia and Reptilia. The consensus of two rounds of whole genome duplication events in early vertebrates and an additional one in early teleosts, along with subsequent recombination events have given rise to many proteins with varying domains and diverse functions in these organisms^32–34^. This can explain the existence of multiple nectins in early teleosts and gnathostomes. Mammals however, do not possess these unique proteins. A similar trend is also seen in case of the HOX family, where teleosts and early vertebrates possess multiple sub-groups, compared to mammalian lineage groups^35^.

Of particular interest, some hits from evolutionarily primitive organisms were found to be proteins with unique domain organizations, not found in any other taxa (Table 2). For example, a protein from Florida lancelet has the following domain organization: IgV/IgC/IgC/TM/SH3. Human nectins are known to bind through their PDZ-binding motif to afadin. Afadin is a multi-domain protein, which in turn binds to actin via its actin-binding domain (which contains a proline-rich segment). Afadin also binds to an SH3-domain-containing protein called ponsin, which is involved in the formation of adherens junction^3,36–38^. SH3 domains are also proline-rich domains, and it could be envisaged that the unique protein present in Florida lancelet might be capable of mediating protein-protein interactions directly through its SH3 domain, thereby avoiding the need for multiple adaptor proteins. Our analysis suggests that primitive organisms may have CAMs that can bypass several steps which are required in higher organisms to mediate cell-cell adhesion, due to the presence of proteins which can carry out more than one of these functions.

A further example of this increase in complexity is as follows. Reports suggest that nectins recruit cadherins to the adherens junctions^2,39^. However, adherens junctions and cadherins predate the nectin family. Previous studies in *Drosophila* have shown that the IgSF protein called echinoid (a protein with 7 Ig-like domains followed by a fibronectin type III domain in the extracellular region) is similar to nectins. Echinoid localizes at the adherens junction, binds to canoe (afadin orthologue in *Drosophila*) and PAR3 (partitioning defective 3 homologue, a protein involved in establishing cell polarity), recruits actin to the junction, and in association with DE-cadherin it helps in cell sorting, morphogenesis and development in *Drosophila*^40^. However, previous studies as well as the current study does not show echinoid as a direct nectin homologue^22^. Although it is difficult to predict the exact functional roles played by these primitive nectin orthologues, it can be hypothesized that the evolution of the nectin family demonstrates the adaptation and integration of newly evolved protein to existing processes, thus leading to more regulation and higher complexity to the processes. The mechanism of cell-cell adhesion is not well explored in early metazoans, thus investigation of these novel proteins with unique domain organizations will provide further insights into currently unknown molecular mechanisms that control cell-cell adhesion.

Considering the fact that nectin family evolved in early chordates, domain organization and functions of these proteins have been conserved with surprising fidelity (Supplementary Table S2). Based on major functions, CAMs show notable family-wise differences in conservation with respect to the number of genes, domain organization, sequence similarity etc. across species. As an example, CAMs involved predominantly in immune regulatory functions are less conserved compared to CAMs involved predominantly in cell-cell adhesion^41–44^. Our study shows that nectins show remarkable sequence conservation across species, as well as negligible gene loss events during metazoan evolution, except for some clades lacking nectin-2 and PVR (both are involved in immune modulatory functions, which can represent adaptations with respect to the immune functions).

While several of the known nectin family interactors are IgSF members, some nectins in fact recognize the same binding partners: for example, nectin-2 and PVR both recognize CD226, TIGIT and nectin-3. Another remarkable example is provided by glycoprotein D of Herpes simplex virus-1, which is capable of recognizing both nectin-1 and nectin-2 as host entry receptors^4^. These nectin-nectin interactions along with the shared interacting partners may have provided a constraint on evolution. The nectin family’s surprising functional and interactome conservation together with negligible loss/gain of genes is an indication of positive evolutionary selective pressure, which in turn hints at the crucial roles performed by these proteins^41,45^.

Cell-cell adhesion is one of the most fundamental steps toward multicellular organization. In addition to providing physical contacts between cells, CAMs mediate and control a plethora of related functions such as cell signalling, proliferation, migration and morphogenesis. Especially, nectin family members are also involved in specific *cis*-interactions with multiple proteins of receptor tyrosine kinase (RTK) family, integrins, etc. involved in cellular signalling pathways^3,6^. Exploring the evolution of these CAMs will help us further our understanding of the underlying mechanisms of CAM-mediated functions, as well as provide insights into the evolution of functionality in protein families.

## Methods

### Multiple sequence alignment of the 9 human nectins

Multiple sequence alignment (MSA) was performed with all 9 human nectins (sequences retrieved from GenBank), using ClustalW. Based on the multiple sequence alignment from ClustalW, an unrooted phylogenetic tree was constructed with PHYLIP package for the 9 human nectins, by neighbour joining method to describe the grouping of the 9 human nectins. Multalin server was used to represent MSA in the results section. Predicted N and O-glycosylation sites were identified using NetNGlyc 1.0 server (http://www.cbs.dtu.dk/services/NetNGlyc/) and NetOGlyc 4.0 server (http://www.cbs.dtu.dk/services/NetOGlyc/), respectively.

### Defining the 9 human nectins

In order to define and identify the sequence characteristics of each of the 9 nectins present in humans, the known orthologues of nectins from a number of vertebrates (wherever possible, because some nectins are absent in some species) were analysed for conserved sequence motifs, characteristics, and domain organizations, after multiple sequence alignment studies.

### Creating blastp hits database for the orthologues of human nectins from the selected organisms

After the initial analyses and identification of characteristics of individual nectin, 39 metazoans representing diverse clades, were first chosen from RefSeq database (https://www.ncbi.nlm.nih.gov/refseq/) (Supplementary Table S1). Full-length amino acid sequences of all 9 members of human nectin family were used as query for blastp search (https://blast.ncbi.nlm.nih.gov/Blast.cgi?PAGE=Proteins) against predicted proteomes of these 39 organisms, retrieved from RefSeq database, to identify nectin family homologues present in each of these organisms. E-value and scores were used to rank the hits. In case of the presence of isoforms, the longest isoform was taken into consideration, in order to avoid ambiguity.

### Analysis of the hits

The following analyses were carried out on each of the top blastp hits for each organism (Supplementary Fig. S3):

#### Reverse-BLAST

The sequence of the top hit was used as query in a fresh blastp search against human proteome, to ascertain the identity of the protein. If a hit gave the same protein from human as the initial query, it was considered a direct orthologue of the initial query. If it gave another of the nectins, then it was considered a paralogue of the original query, with the original query protein being absent in the organism.

#### Domain search

The domain organization of each of the hits were analysed with the following online servers: SMART (Simple Modular Architecture Research Tool)^46^, InterProScan^47^, CDD (Conserved Domain Database)^48^ and TMHMM (Transmembrane Hidden Markov Models)^49^. Multiple databases were used in order to cross-check the results.

## Supplementary information

Supplementary information.
