# Supplementary material for: Tracing the evolution of nectin and nectin-like cell adhesion molecules
Source: Sci Rep. 2020 Jun 10;10:9434. doi: 10.1038/s41598-020-66461-4 (PMC7286890; doi:10.1038/s41598-020-66461-4)
Supplement: Supplementary file 1 — Supplementary information. [file 41598_2020_66461_MOESM1_ESM.pdf]

SUPPLEMENTARY INFORMATION

Tracing the evolution of nectin and nectin-like cell adhesion molecules

Kheerthana Duraivelan[1,*] and Dibyendu Samanta[1,*]

[1]School of Bioscience, Indian Institute of Technology Kharagpur, Kharagpur-721302, West Bengal, India.

*To whom correspondence should be addressed:

Dibyendu Samanta, School of Bioscience, Sir J. C. Bose Laboratory Complex, Indian Institute of Technology Kharagpur, Kharagpur 721302, West Bengal, India; Phone: (03222) 260295

E-mail: dibyendu.samanta@iitkgp.ac.in

Kheerthana Duraivelan, School of Bioscience, Sir J. C. Bose Laboratory Complex, Indian Institute of Technology Kharagpur, Kharagpur 721302, West Bengal, India.

E-mail: kheerthanaduraivelan@iitkgp.ac.in

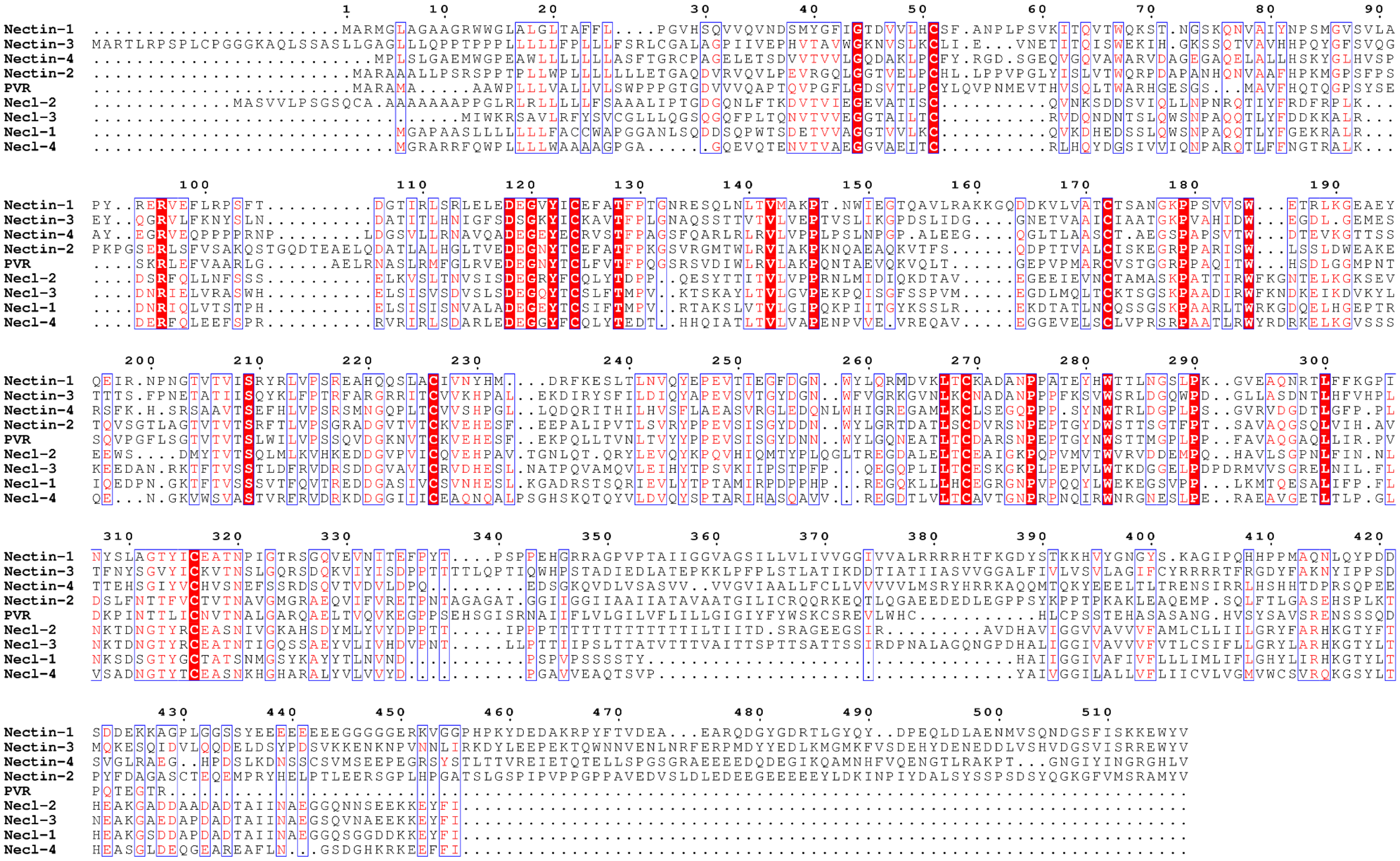

Supplementary Figure S1: Multiple sequence alignment of all 9 human nectins. The sequences of all 9 human nectin homologues were aligned with Multalin. Although the results show some conserved residues (highlighted in red), nectins and nectin-likes actually show differences in their sequences, and form 2 different sub-groups within the nectin family.

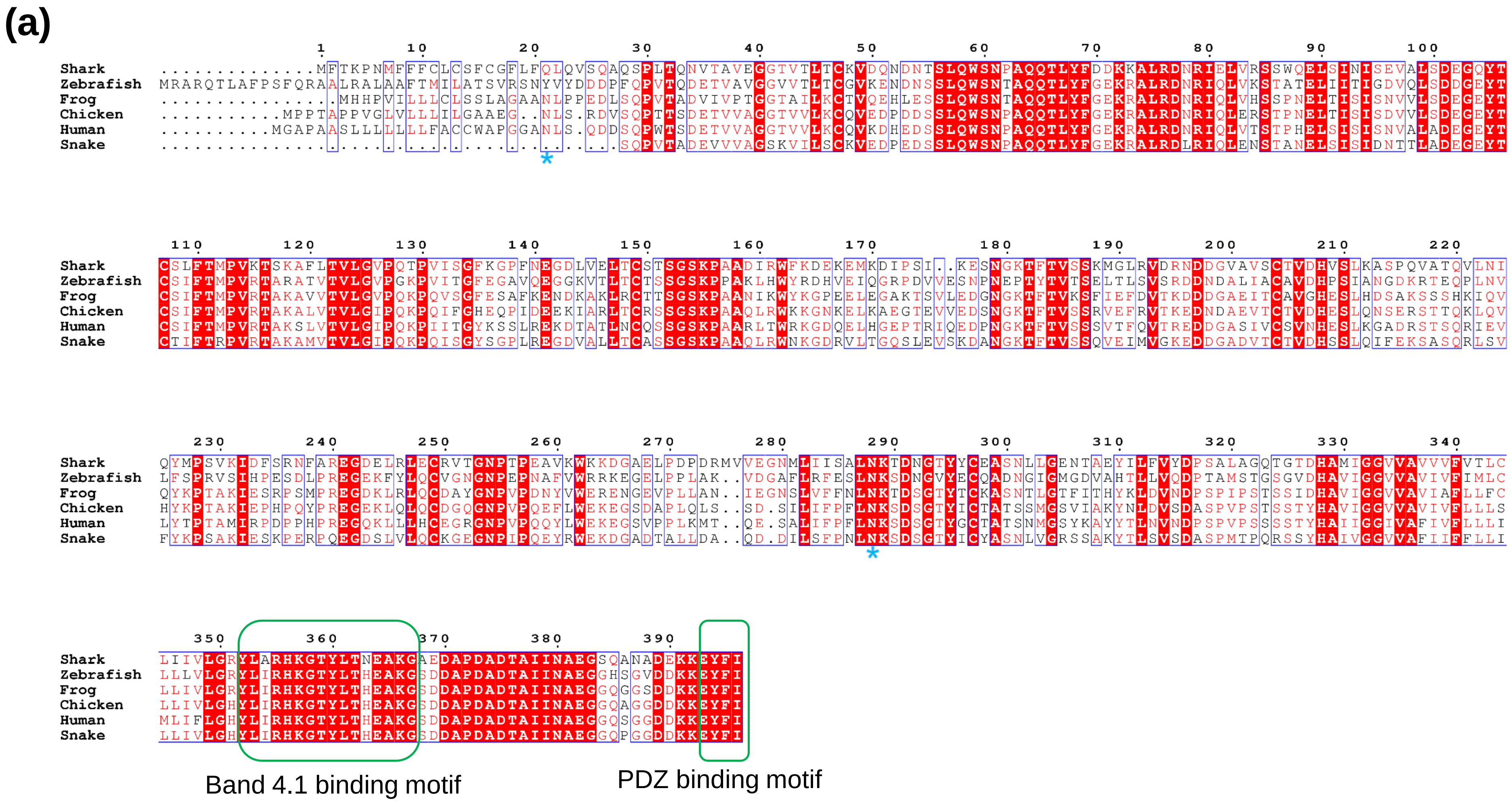

Supplementary Figure S2: Multiple sequence alignment of individual nectin family members from different organisms. The sequences of nectin and nectin-like orthologues were aligned with Multalin. (a) Necl-1, (b) Necl-2, (c) Necl-3, (d) Necl-4, (e) Nectin-1, (f) Nectin-2, (g) Nectin-3, (h) Nectin-4 and (i) PVR. * and 0 denote predicted N-linked and O-linked glycosylation sites respectively.

(b)

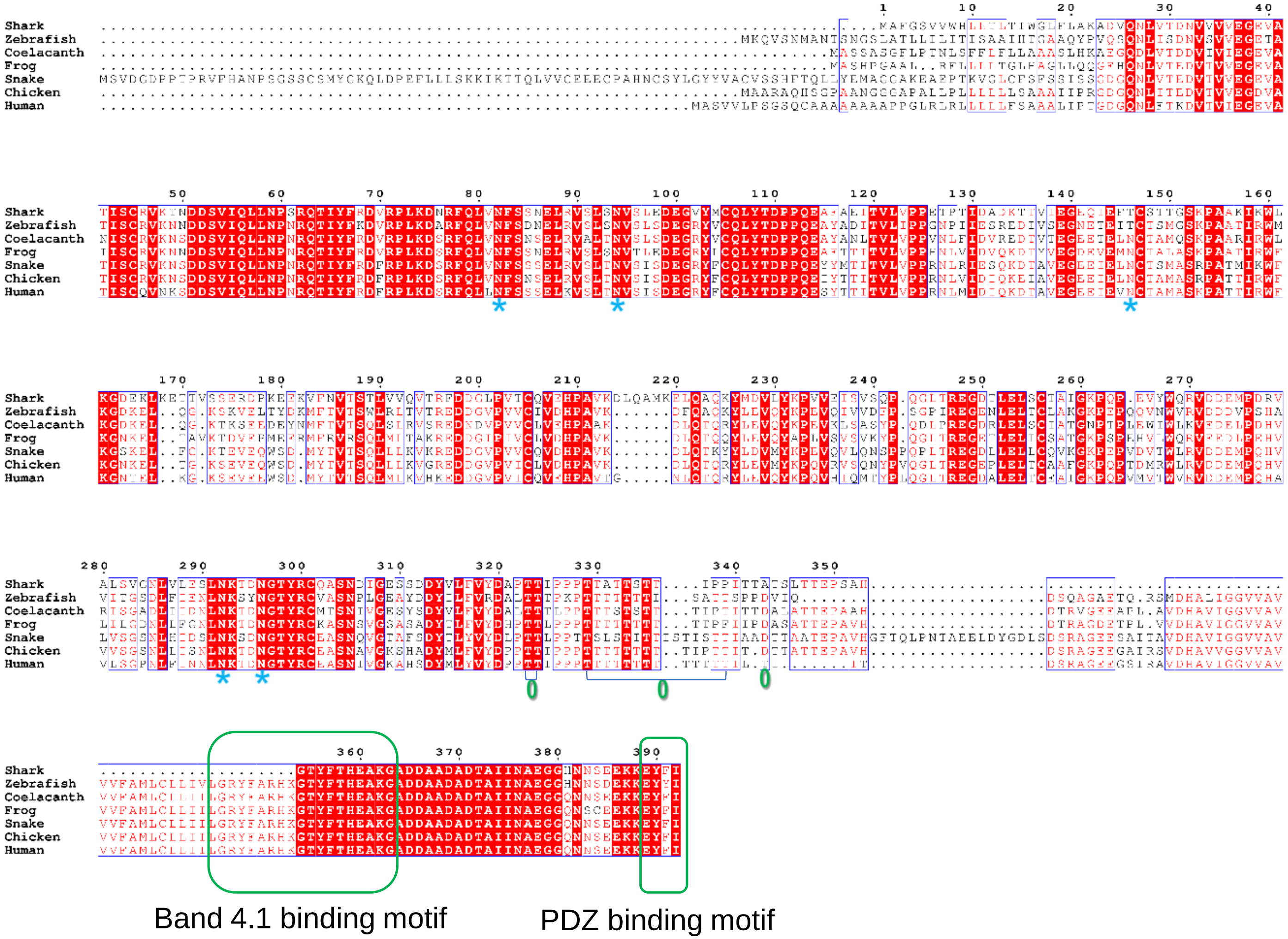
Shark
Zebrafish
Coelacanth
Frog
Snake
Chicken
Human
Band 4.1 binding motif
PDZ binding motif

(c)

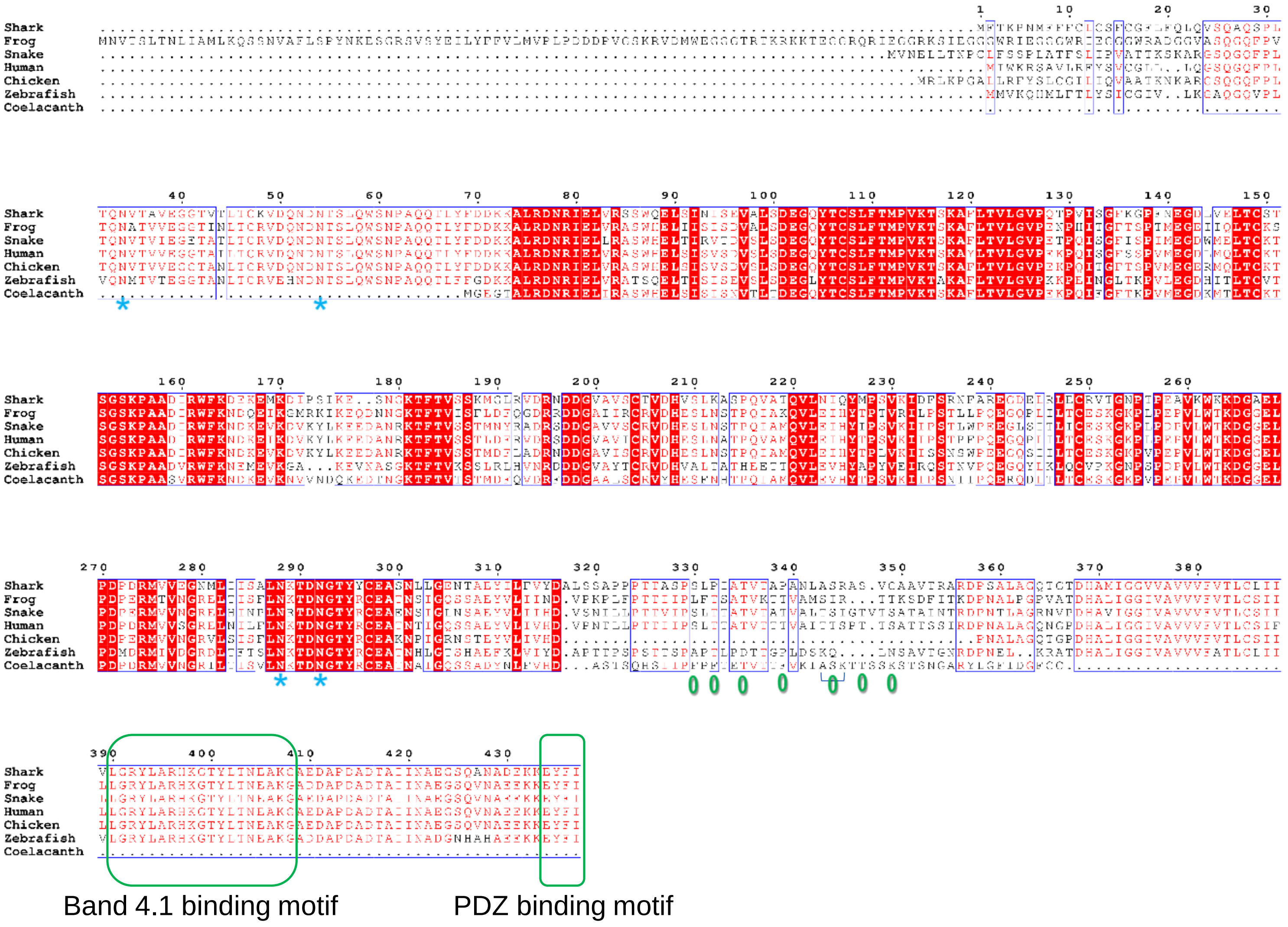
Shark
Frog
Snake
Human
Chicken
Zebrafish
Coelacanth
Band 4.1 binding motif
PDZ binding motif

(d)

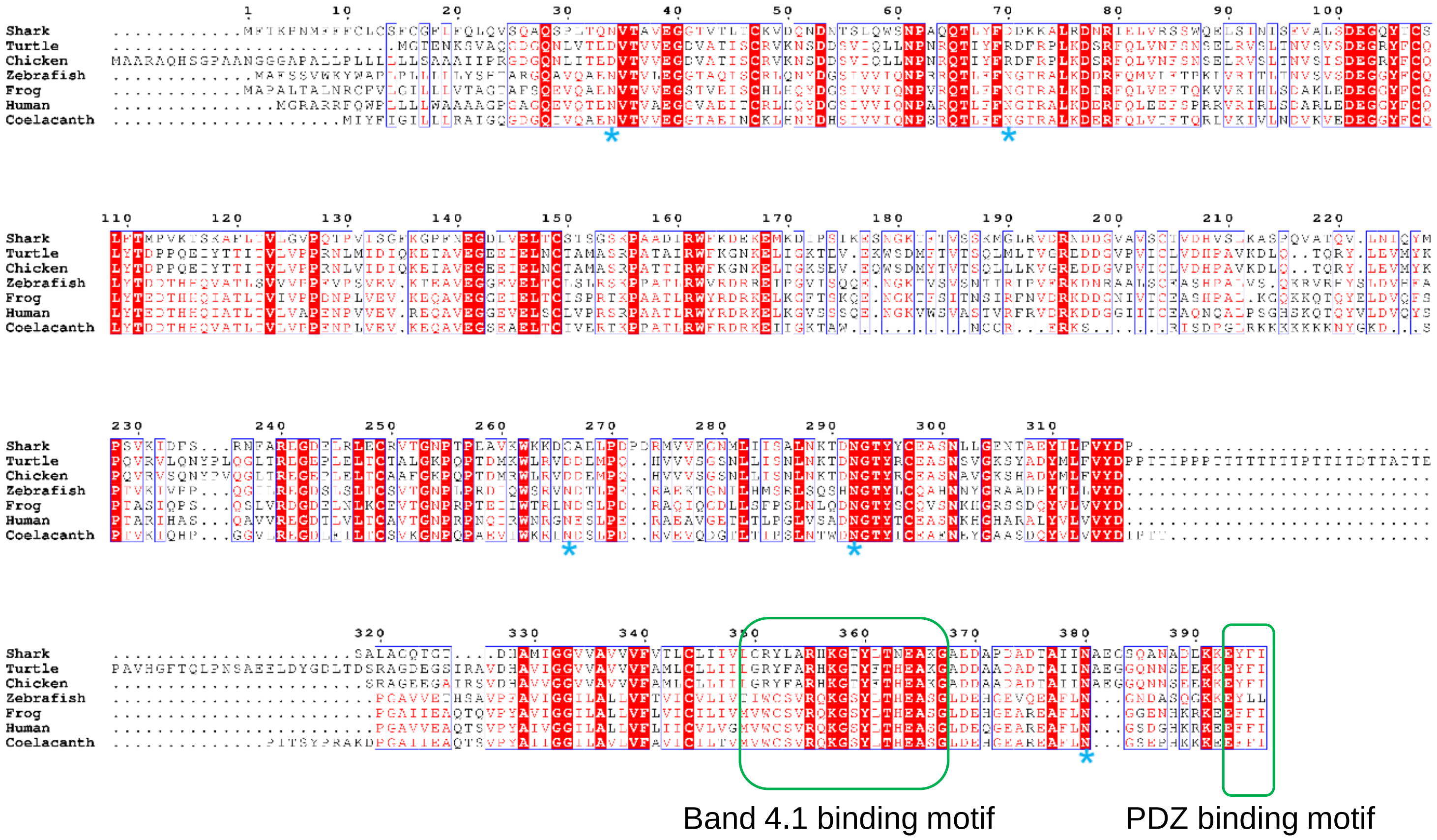
Shark
Turtle
Chicken
Zebrafish
Frog
Human
Coelacanth
Band 4.1 binding motif
PDZ binding motif

(e)

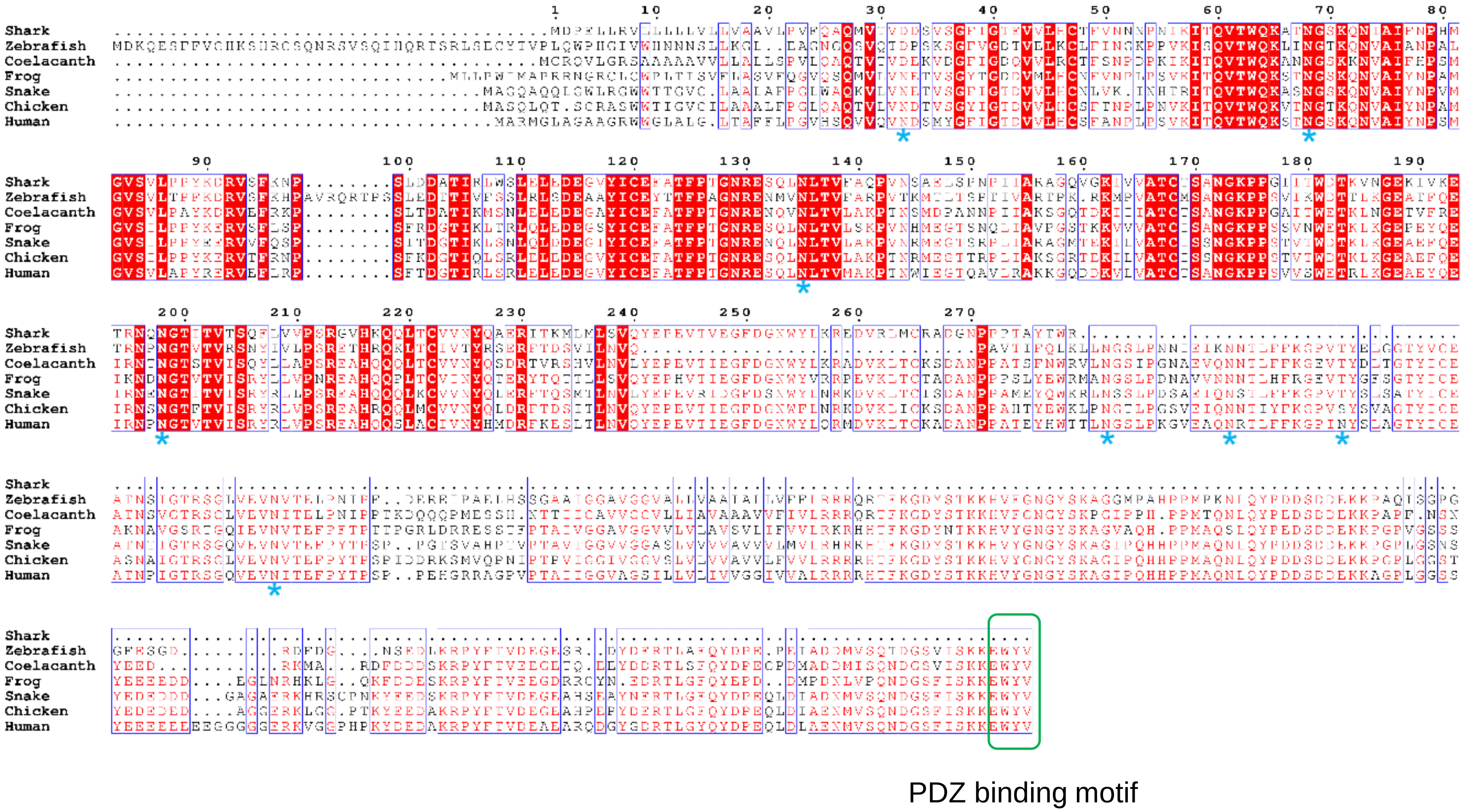
Shark
Zebrafish
Coelacanth
Frog
Snake
Chicken
Human
PDZ binding motif

(f)

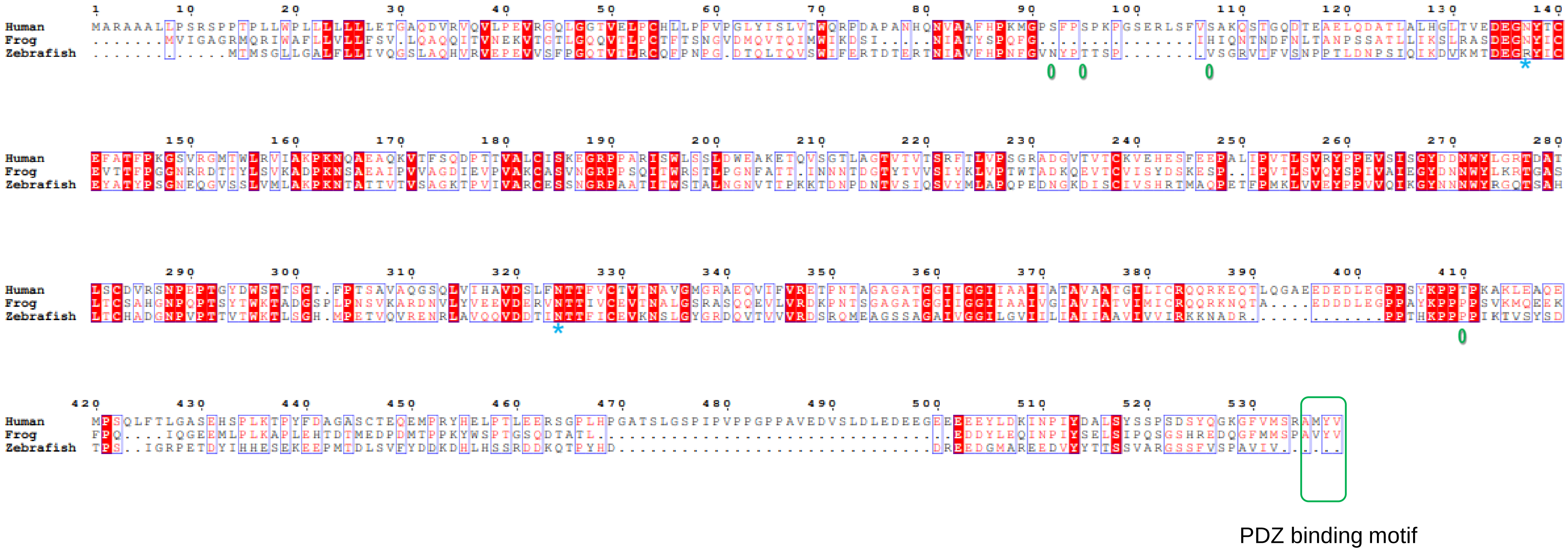

(g)

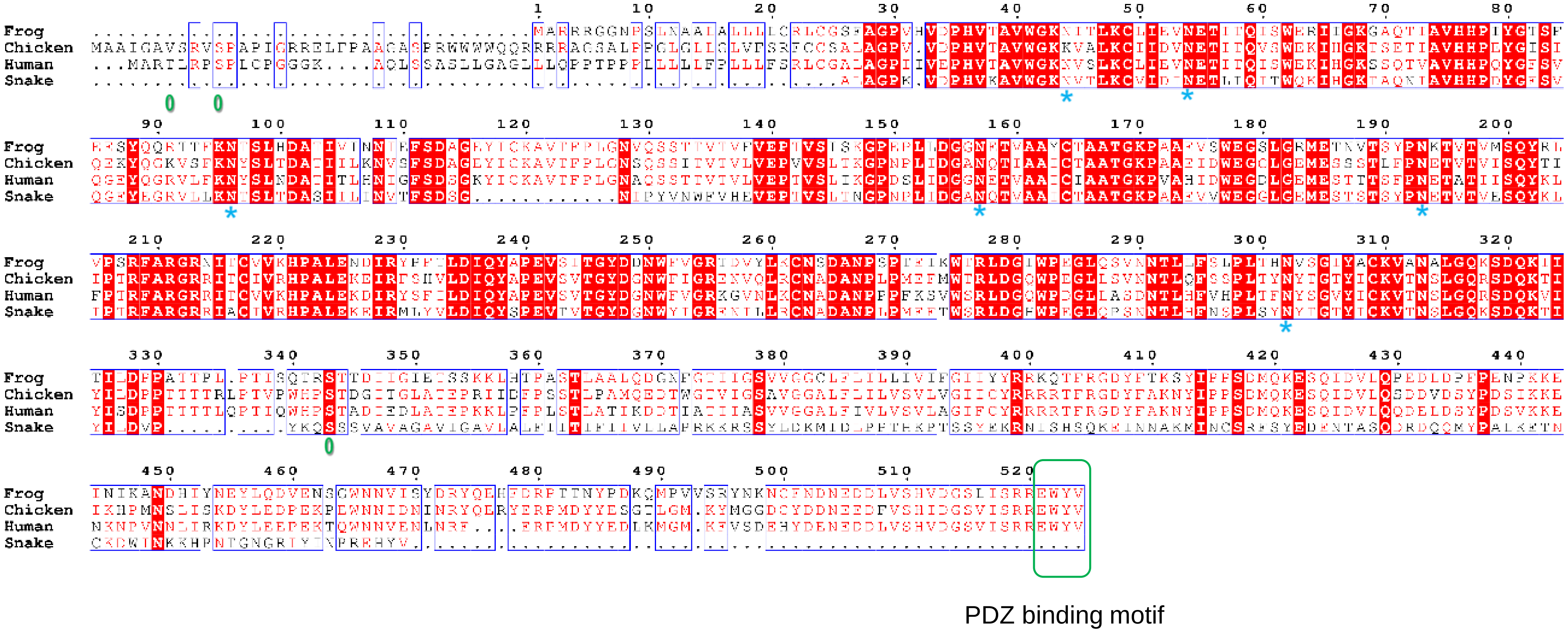

(h)

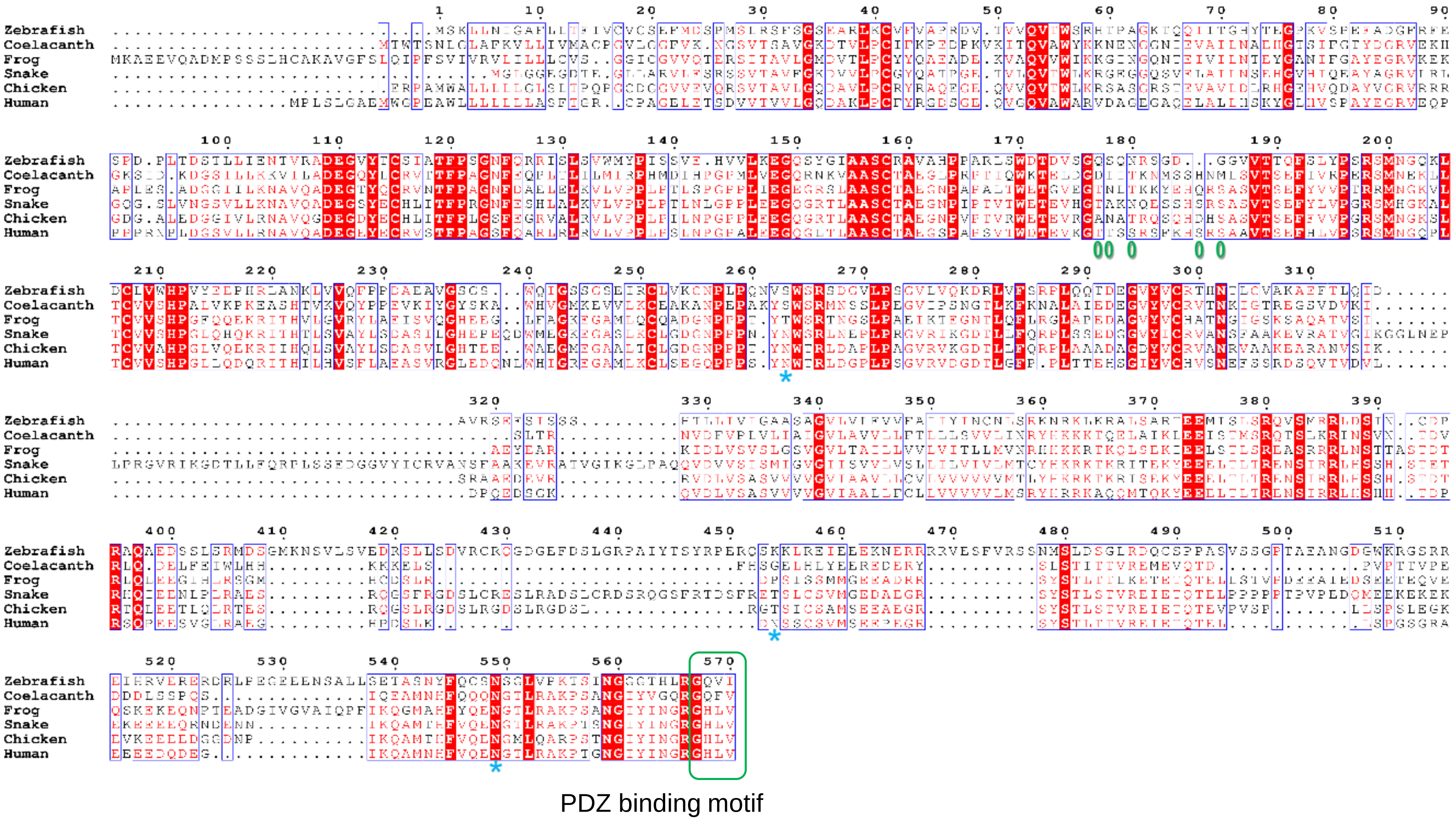
Zebrafish
Coelacanth
Frog
Snake
Chicken
Human
PDZ binding motif

(i)

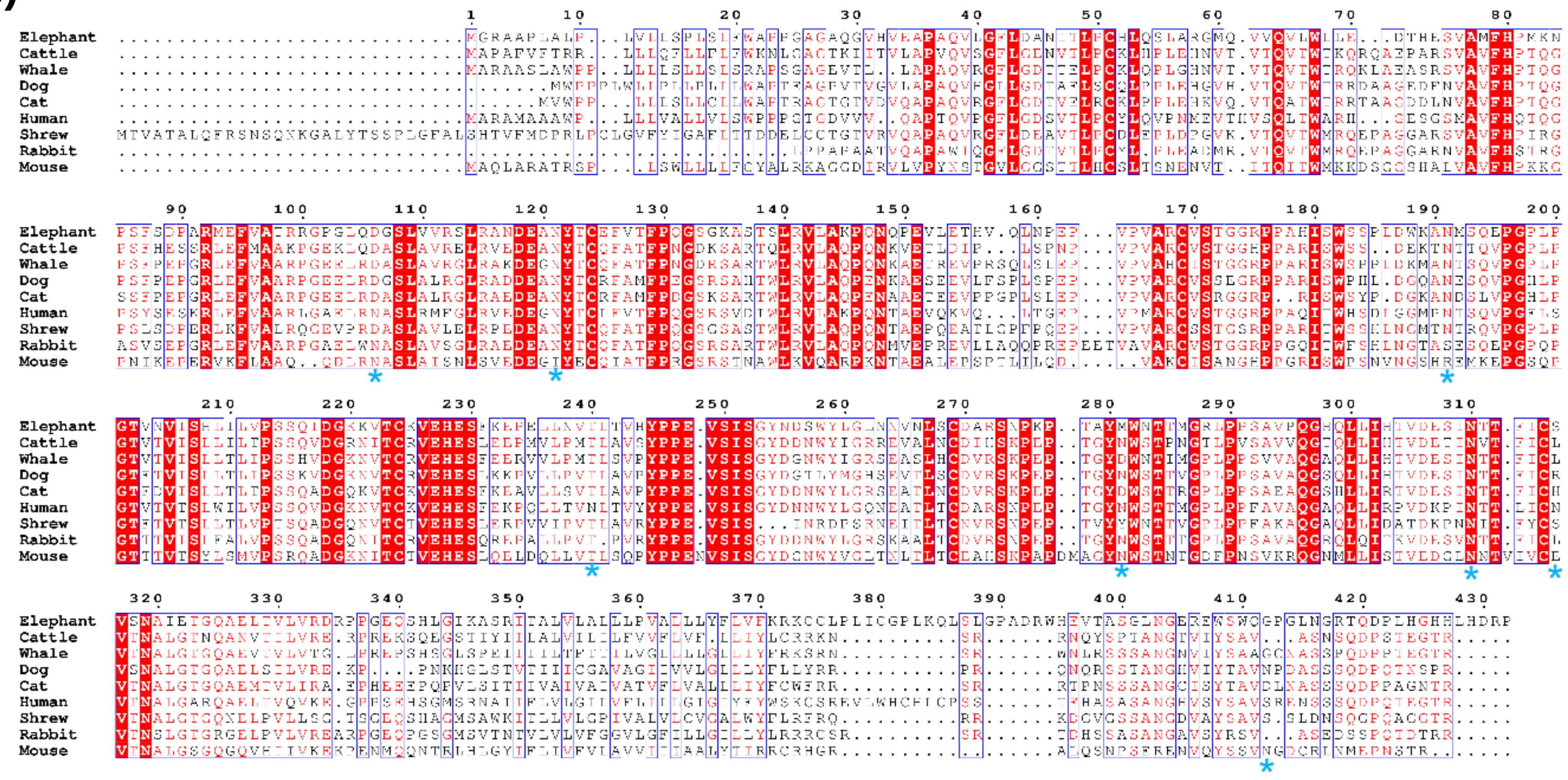

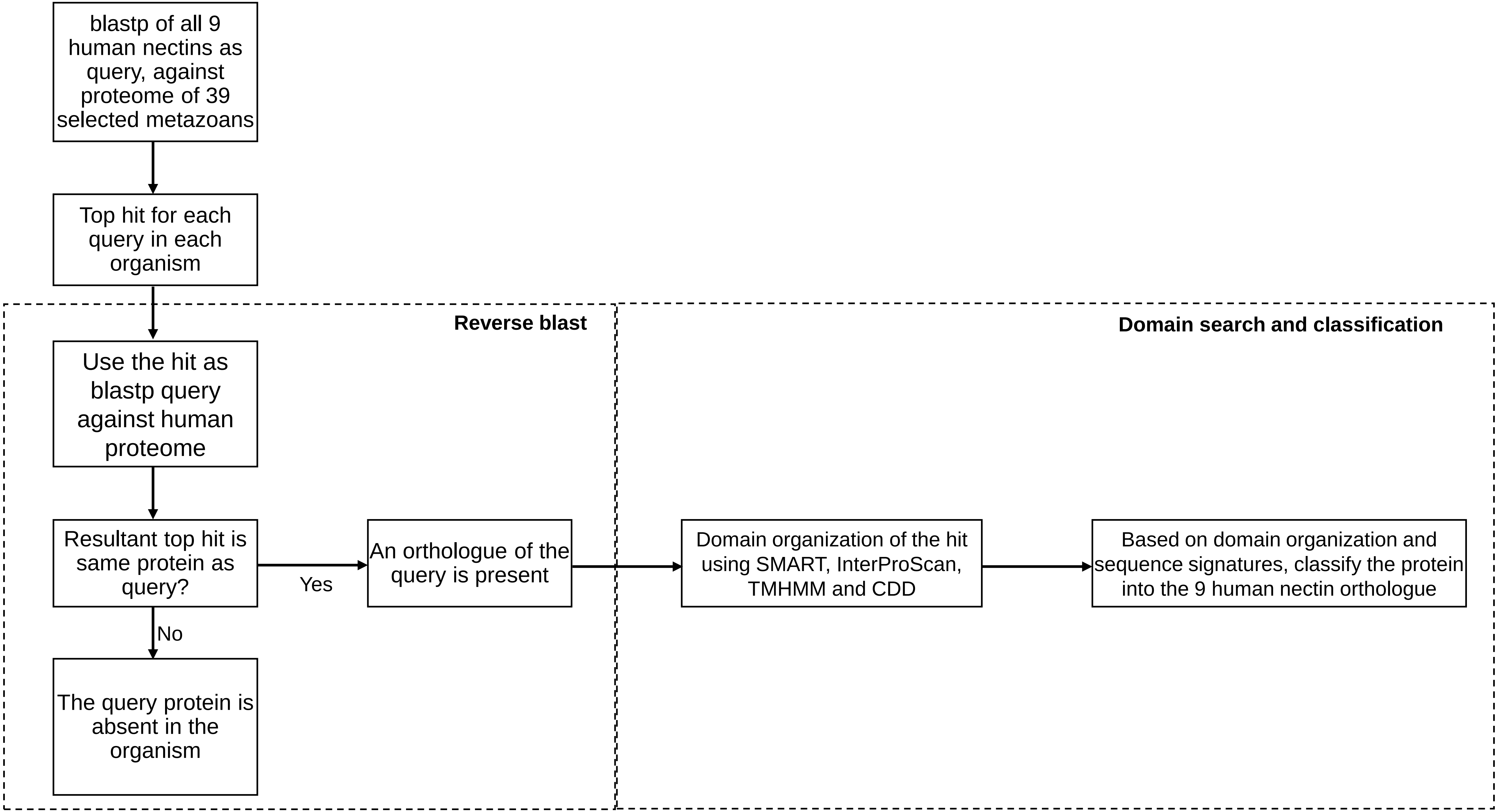

Supplementary Figure S3: Analysis of the hits. The work flow for the analysis is described in this figure.

SUPPLEMENTARY TABLES

Supplementary Table 1: List of organisms considered for the study

Sl.no.	Organism	Common name	Name used in figure	Organism id
1	*Acanthaster planci*	Crown-of-thorns starfish	Starfish	133434
2	*Alligator mississippiensis*	American alligator	Alligator	8496
3	*Amphimedon queenslandica*	Sponge	Sponge	400682
4	*Anopheles gambiae*	African malaria mosquito	Mosquito	7165
5	*Apis mellifera*	Honey bee	Bee	7460
6	*Bombyx mori*	Domestic silkworm	Silk worm	7091
7	*Bos taurus*	Cattle	Cow	9913
8	*Branchiostoma floridae*	Florida lancelet	Lancet	7739
9	*Caenorhabditis elegans*	-	*C. elegans*	6239
10	*Callorhinchus milii*	Elephant shark	Shark	7868
11	*Canis lupus familiaris*	Dog	Dog	9615
12	*Ciona intestinalis*	Vase tunicate/Sea squirt	Sea squirt	7719
13	*Crassostrea gigas*	Pacific oyster	Oyster	29159
14	*Danio rerio*	Zebrafish	Zebrafish	7955
15	*Drosophila melanogaster*	Fruit fly	Fly	7227
16	*Felis catus*	Domestic cat	Cat	9685
17	*Gallus gallus*	Chicken	Chicken	9031
18	*Helobdella robusta*	Leech	Leech	6412
19	*Homo sapiens*	Human	Human	9606
20	*Hydra vulgaris*	Hydra	Hydra	6087
21	*Latimeria chalumnae*	Coelacanth	Coelacanth	7897
22	*Lingula anatina*	Lamp shell	Lamp shell	7574
23	*Loxodonta africana*	African savanna elephant	Elephant	9785
24	*Mus musculus*	House mouse	Mouse	10090
25	*Myotis brandtii*	Brandt's bat	Bat	109478
26	*Nematostella vectensis*	Starlet sea anemone	Sea anemone	45351
27	*Orcinus orca*	Killer whale	Orca	9733
28	*Ornithorhynchus anatinus*	Platypus	Platypus	9258
29	*Oryctolagus cuniculus*	Rabbit	Rabbit	9986
30	*Pelodiscus sinensis*	Chinese soft-shelled turtle	Turtle	13735
31	*Protobothrops mucrosquamatus*	Pit viper	Pit viper	103944
32	*Saccoglossus kowalevskii*	Acorn worm	Acorn worm	10224
33	*Sarcophilus harrisii*	Tasmanian devil	Tasmanian devil	9305
34	*Schistosoma mansoni*	Blood fluke	Blood fluke	6183
35	*Scleropages formosus*	Asian bonytongue	Bonytongue	113540
36	*Strongylocentrotus purpuratus*	Purple sea urchin	Sea urchin	7668
37	*Stylophora pistillata*	Stony corals	Coral	50429
38	*Tupaia chinensis*	Chinese tree shrew	Shrew	246437
39	*Xenopus laevis*	African clawed frog	Frog	8355

Supplementary Table 2: Top hits and corresponding analyses

Organism	Description	Nectin-1	Nectin-2	Nectin-3	Nectin-4	CADM-3/Necl-1	CADM-1/Necl-2	CADM-2/Necl-3	CADM-4	PVR
H.sapiens	RefSeq protein name	Nectin-1	Nectin-2	Nectin-3	Nectin-4	CADM-3	CADM-1	CADM-2	CADM-4	PVR
	RefSeq protein id	NP_002846.3	NP_001036189.1	NP_056295.1	NP_112178.2	NP_001120645.1	NP_055148.3	XP_006713144.1	NP_660339.1	NP_006496.4
	Reverse BLAST result	Nectin-1	Nectin-2	Nectin-3	Nectin-4	Necl-1	Necl-2	Necl-3	Necl-4	PVR
	Hit identified as	Nectin-1	Nectin-2	Nectin-3	Nectin-4	Necl-1	Necl-2	Necl-3	Necl-4	PVR
	Domain organization	Ig/Ig/Ig/TM	Ig/Ig/Ig/TM	Ig/Ig/Ig/TM	Ig/Ig/Ig/TM	Ig/Ig/Ig/TM/b4.1bm	Ig/Ig/Ig/TM/b4.1bm	Ig/Ig/Ig/TM/b4.1bm	Ig/Ig/Ig/TM/b4.1bm	Ig/Ig/Ig/TM
T.chinensis	RefSeq protein name	Nectin-1	Nectin-2	Nectin-3	Nectin-4	CADM-3	CADM-1	CADM-2	CADM-4	PVR
	RefSeq protein id	XP_006152381.1	XP_006142214.2	XP_006155211.1	XP_006163166.1	XP_006168922.1	XP_014438306.1	XP_006159832.1	XP_006142091.1	XP_006142209.1

	Reverse BLAST result	Nectin-1	Nectin-2	Nectin-3	Nectin-4	Necl-1	Necl-2	Necl-3	Necl-4	PVR
	Hit identified as	Nectin-1	Nectin-2	Nectin-3	Nectin-4	Necl-1	Necl-2	Necl-3	Necl-4	PVR
	Domain organization	Ig/Ig/Ig/TM	Ig/Ig/Ig/TM	Ig/Ig/Ig/TM	Ig/Ig/Ig/TM	Ig/Ig/Ig/TM/b4.1bm	Ig/Ig/Ig/TM/b4.1bm	Ig/Ig/Ig/TM/b4.1bm	Ig/Ig/Ig/TM/b4.1bm	Ig/Ig/Ig/TM
O.cuniculus	RefSeq protein name	Nectin-1	Nectin-2	Nectin-3	Nectin-4	CADM-3	CADM-1	CADM-2	CADM-4	PVR
	RefSeq protein id	XP_008265430.1	XP_008250475.1	XP_008265188.1	XP_002715262.1	XP_002715327.2	XP_002708453.1	XP_008265326.2	XP_017194603.1	NP_001164588.1
	Reverse BLAST result	Nectin-1	Nectin-2	Nectin-3	Nectin-4	Necl-1	Necl-2	Necl-3	Necl-4	PVR
	Hit identified as	Nectin-1	Nectin-2	Nectin-3	Nectin-4	Necl-1	Necl-2	Necl-3	Necl-4	PVR
	Domain organization	Ig/Ig/Ig/TM	Ig/Ig/Ig/TM	Ig/Ig/Ig/TM	Ig/Ig/Ig/TM	Ig/Ig/Ig/TM/b4.1bm	Ig/Ig/Ig/TM/b4.1bm	Ig/Ig/Ig/TM/b4.1bm	Ig/Ig/Ig/TM/b4.1bm	Ig/Ig/Ig/TM
M.musculus	RefSeq protein name	Nectin-1	Nectin-2	Nectin-3	Nectin-4	CADM-3	CADM-1	CADM-2	CADM-4	PVR

	RefSeq protein id	NP_067399.2	NP_033016.3	NP_067470.1	NP_082169.2	NP_444429.1	NP_061240.3	XP_006523091.1	NP_694752.1	NP_081790.1
	Reverse BLAST result	Nectin-1	Nectin-2	Nectin-3	Nectin-4	Necl-1	Necl-2	Necl-3	Necl-4	PVR
	Hit identified as	Nectin-1	Nectin-2	Nectin-3	Nectin-4	Necl-1	Necl-2	Necl-3	Necl-4	PVR
	Domain organization	Ig/Ig/Ig/TM	Ig/Ig/Ig/TM	Ig/Ig/Ig/TM	Ig/Ig/Ig/TM	Ig/Ig/Ig/TM/b4.1bm	Ig/Ig/Ig/TM/b4.1bm	Ig/Ig/Ig/TM/b4.1bm	Ig/Ig/Ig/TM/b4.1bm	Ig/Ig/Ig/TM
O.orca	RefSeq protein name	Nectin-1	Nectin-2	Nectin-3	Nectin-4	CADM-3	CADM-1	CADM-2	CADM-4	PVR
	RefSeq protein id	XP_004273339.1	XP_004271237.1	XP_004272121.1	XP_004284464.1	XP_004284516.1	XP_004273421.1	XP_004272698.1	XP_004271256.1	XP_012388979.1
	Reverse BLAST result	Nectin-1	Nectin-2	Nectin-3	Nectin-4	Necl-1	Necl-2	Necl-3	Necl-4	PVR
	Hit identified as	Nectin-1	Nectin-2	Nectin-3	Nectin-4	Necl-1	Necl-2	Necl-3	Necl-4	PVR
	Domain organization	Ig/Ig/Ig/TM	Ig/Ig/Ig/TM	Ig/Ig/Ig/TM	Ig/Ig/Ig/TM	Ig/Ig/Ig/TM/b4.1bm	Ig/Ig/Ig/TM/b4.1bm	Ig/Ig/Ig/TM/b4.1bm	Ig/Ig/Ig/TM/b4.1bm	Ig/Ig/Ig/TM

B.taurus	RefSeq protein name	poliovirus receptor-related protein 1	Nectin-2	Nectin-3	Nectin-4	CADM-3	CADM-1	CADM-2	CADM-4	PVR
	RefSeq protein id	NP_001192951.1	XP_005219253.1	XP_024850003.1	XP_010801051.1	NP_001069414.1	XP_005215903.1	XP_010799264.1	XP_024834910.1	XP_005219484.2
	Reverse BLAST result	Nectin-1	Nectin-2	Nectin-3	Nectin-4	Necl-1	Necl-2	Necl-3	Necl-4	PVR
	Hit identified as	Nectin-1	Nectin-2	Nectin-3	Nectin-4	Necl-1	Necl-2	Necl-3	Necl-4	PVR
	Domain organization	Ig/Ig/Ig/TM	Ig/Ig/Ig/TM	Ig/Ig/Ig/TM	Ig/Ig/Ig/TM	Ig/Ig/Ig/TM/b4.1bm	Ig/Ig/Ig/TM/b4.1bm	Ig/Ig/Ig/TM/b4.1bm	Ig/Ig/Ig/TM/b4.1bm	Ig/Ig/Ig/TM
F.catus	RefSeq protein name	Nectin-1	Nectin-2	Nectin-3	Nectin-4	CADM-3	CADM-1	CADM-2	CADM-4	PVR
	RefSeq protein id	XP_003992493.1	XP_003997741.4	XP_023115886.1	XP_019677856.2	XP_023103769.1	XP_023094894.1	XP_003991586.1	XP_023101118.1	XP_003997776.2
	Reverse BLAST result	Nectin-1	Nectin-2	Nectin-3	Nectin-4	Necl-1	Necl-2	Necl-3	Necl-4	PVR
	Hit identified as	Nectin-1	Nectin-2	Nectin-3	Nectin-4	Necl-1	Necl-2	Necl-3	Necl-4	PVR

	Domain organization	Ig/Ig/Ig/TM	Ig/Ig/Ig/TM	Ig/Ig/Ig/TM	Ig/Ig/Ig/TM	Ig/Ig/Ig/TM/b4.1bm	Ig/Ig/Ig/TM/b4.1bm	Ig/Ig/Ig/TM/b4.1bm	Ig/Ig/Ig/TM/b4.1bm	Ig/Ig/Ig/TM
C.familiaris	RefSeq protein name	Nectin-1	Nectin-2	Nectin-3	Nectin-4	CADM-3	CADM-1	CADM-2	CADM-4	PVR
	RefSeq protein id	NP_001274057.1	XP_852651.1	XP_022269668.1	NP_001300782.1	XP_013966732.1	XP_022273988.1	XP_544804.3	XP_541580.3	XP_005616524.1
	Reverse BLAST result	Nectin-1	Nectin-2	Nectin-3	Nectin-4	Necl-1	Necl-2	Necl-3	Necl-4	PVR
	Hit identified as	Nectin-1	Nectin-2	Nectin-3	Nectin-4	Necl-1	Necl-2	Necl-3	Necl-4	PVR
	Domain organization	Ig/Ig/Ig/TM	Ig/Ig/Ig/TM	Ig/Ig/Ig/TM	Ig/Ig/Ig/TM	Ig/Ig/Ig/TM/b4.1bm	Ig/Ig/Ig/TM/b4.1bm	Ig/Ig/Ig/TM/b4.1bm	Ig/Ig/Ig/TM/b4.1bm	Ig/Ig/Ig/TM
M.brandtii	RefSeq protein name	Nectin-1	Nectin-2	Nectin-3	Nectin-4	CADM-3	CADM-1	CADM-2	CADM-4	Nectin-2
	RefSeq protein id	XP_005856821.1	XP_005863453.1	XP_014396719.1	XP_014384032.1	XP_005884868.2	XP_005856961.1	XP_005878157.1	XP_005886583.1	XP_005863454.2
	Reverse BLAST result	Nectin-1	Nectin-2	Nectin-3	Nectin-4	Necl-1	Necl-2	Necl-3	Necl-4	Nectin-2

	Hit identified as	Nectin-1	Nectin-2	Nectin-3	Nectin-4	Necl-1	Necl-2	Necl-3	Necl-4	
	Domain organization	Ig/Ig/Ig/TM	Ig/Ig/Ig/TM	Ig/Ig/Ig/TM	Ig/Ig/Ig/TM	Ig/Ig/Ig/TM/b4.1bm	Ig/Ig/Ig/TM/b4.1bm	Ig/Ig/Ig/TM/b4.1bm	Ig/Ig/Ig/TM/b4.1bm	Ig/Ig/Ig/TM
L.africana	RefSeq protein name	Nectin-1	Nectin-2	Nectin-3	Nectin-4	CADM-3	CADM-1	CADM-2	CADM-4	PVR homolog
	RefSeq protein id	XP_023413980.1	XP_010585209.1	XP_023396174.1	XP_010593186.2	XP_023409920.1	XP_003418271.1	XP_010588676.1	XP_023399792.1	XP_023398920.1
	Reverse BLAST result	Nectin-1	Nectin-2	Nectin-3	Nectin-4	Necl-1	Necl-2	Necl-3	Necl-4	PVR
	Hit identified as	Nectin-1	Nectin-2	Nectin-3	Nectin-4	Necl-1	Necl-2	Necl-3	Necl-4	PVR
	Domain organization	Ig/Ig/Ig/TM	Ig/Ig/Ig/TM	Ig/Ig/Ig/TM	Ig/Ig/Ig/TM	Ig/Ig/Ig/TM/b4.1bm	Ig/Ig/Ig/TM/b4.1bm	Ig/Ig/Ig/TM/b4.1bm	Ig/Ig/Ig/TM/b4.1bm	Ig/Ig/Ig/TM
S.harrisii	RefSeq protein name	Nectin-1	Nectin-2	Nectin-3	Nectin-4	CADM-3	CADM-1	CADM-2	CADM-4-like [Partial]	Nectin-2
	RefSeq protein id	XP_012400386.1	XP_012401050.2	XP_031817139.1	XP_012404940.1	XP_003767933.1	XP_023355211.1	XP_003765566.1	XP_012402159.1	XP_012401050.2

	Reverse BLAST result	Nectin-1	Nectin-2	Nectin-3	Nectin-4	Necl-1	Necl-2	Necl-3	Necl-4	Nectin-2
	Hit identified as	Nectin-1	Nectin-2	Nectin-3	Nectin-4	Necl-1	Necl-2	Necl-3	Necl-4	
	Domain organization	Ig/Ig/Ig/TM	Ig/Ig/Ig/TM	Ig/Ig/Ig/TM	Ig/Ig/Ig/TM	Ig/Ig/Ig/TM/b4.1bm	Ig/Ig/Ig/TM/b4.1bm	Ig/Ig/Ig/TM/b4.1bm	Ig/Ig	Ig/Ig/Ig/TM
O.anatinus	RefSeq protein name	Nectin-1	Nectin-2-like [Partial]	Nectin-3 [Partial]	Nectin-4 [Partial]	CADM-2 [Partial]	CADM-1-like	CADM-2 [Partial]	CADM-4 [Partial]	Nectin-2-like [Partial]
	RefSeq protein id	XP_007666212.1	XP_007661562.1	XP_001509965.2	XP_001509463.1	XP_007664822.1	XP_007653982.1	XP_007664821.1	XP_007661610.1	XP_007661562.1
	Reverse BLAST result	Nectin-1	Nectin-2	Nectin-3	Nectin-4	Necl-3	Necl-2	Necl-3	Necl-4	Nectin-2
	Hit identified as	Nectin-1	Nectin-2	Nectin-3	Nectin-4		Necl-2	Necl-3	Necl-4	
	Domain organization	Ig/Ig/Ig/TM	Ig/Ig/Ig/TM	Ig/Ig/TM	Ig/Ig/TM	Ig/Ig/TM/b4.1bm	TM/Ig/Ig/Ig	Ig/Ig/TM/b4.1bm	Ig/Ig/Ig	Ig/Ig/Ig/TM
G.gallus	RefSeq protein name	Nectin-1	Nectin-1	Nectin-3	Nectin-4	CADM-3	CADM-1	CADM-2	CADM-1	Nectin-1

	RefSeq protein id	XP_024999006.1	XP_024999007.1	XP_025000020.1	NP_001305355.1	XP_015154160.1	XP_015153705.1	XP_015153881.1	XP_015153709.1	XP_024999007.1
	Reverse BLAST result	Nectin-1	Nectin-1	Nectin-3	Nectin-4	Necl-1	Necl-2	Necl-3	Necl-2	Nectin-1
	Hit identified as	Nectin-1		Nectin-3	Nectin-4	Necl-1	Necl-2	Necl-3		
	Domain organization	Ig/Ig/Ig/TM	Ig/Ig/Ig/TM	Ig/Ig/Ig/TM	Ig/Ig/Ig/TM	Ig/Ig/Ig/TM/b4.1bm	Ig/Ig/Ig/TM/b4.1bm	Ig/Ig/Ig/TM/b4.1bm	Ig/Ig/Ig/TM/b4.1bm	Ig/Ig/Ig/TM
A.mississippiensis	RefSeq protein name	Nectin-1	Nectin-2	Nectin-3-like (Low quality protein)	Nectin-4	CADM-3	CADM-1	CADM-2	CADM-4	Nectin-2
	RefSeq protein id	XP_006258219.1	XP_019333053.1	XP_019353385.1	XP_019353166.1	XP_019351079.1	XP_019336211.1	XP_019331993.1	XP_014456714.1	XP_019333053.1
	Reverse BLAST result	Nectin-1	Nectin-2	Nectin-3	Nectin-4	Necl-1	Necl-2	Necl-3	Necl-4	Nectin-2
	Hit identified as	Nectin-1	Nectin-2	Nectin-3	Nectin-4	Necl-1	Necl-2	Necl-3	Necl-4	
	Domain organization	Ig/Ig/Ig/TM	Ig/Ig/Ig/TM	Ig/Ig/Ig/TM	Ig/Ig/Ig/TM	Ig/Ig/Ig/TM/b4.1bm	Ig/Ig/Ig/TM/b4.1bm	Ig/Ig/Ig/TM/b4.1bm	Ig/Ig/Ig/TM/b4.1bm	Ig/Ig/Ig/TM

P.sinensis	RefSeq protein name	Nectin-1 [Low quality protein]	Nectin-1	Nectin-3	Nectin-4 [Low quality protein]	CADM-3	CADM-1	CADM-2	CADM-1	Nectin-1
	RefSeq protein id	XP_014426036.1	XP_014426036.1	XP_014425827.1	XP_006115272.2	XP_025036537.1	XP_025039718.1	XP_006110859.1	XP_025039718.1	XP_014426036.1
	Reverse BLAST result	Nectin-1	Nectin-1	Nectin-3	Nectin-4	Necl-1	Necl-2	Necl-3	Necl-2	Nectin-1
	Hit identified as	Nectin-1		Nectin-3	Nectin-4	Necl-1	Necl-2	Necl-3		
	Domain organization	Ig/Ig/Ig/TM	Ig/Ig/Ig/TM	Ig/Ig/Ig/TM	Ig/Ig/Ig/TM	Ig/Ig/Ig/TM/b4.1bm	Ig/Ig/Ig/TM/b4.1bm	Ig/Ig/Ig/TM/b4.1bm	Ig/Ig/Ig/TM/b4.1bm	Ig/Ig/Ig/TM
P.mucrosquamatus	RefSeq protein name	Nectin-1	Nectin-1-like	Nectin-3	Nectin-4	CADM-3	CADM-1	CADM-2	CADM-4 [Partial]	Nectin-1-like
	RefSeq protein id	XP_015669049.1	XP_015682671.1	XP_015681470.1	XP_015680321.1	XP_015683338.1	XP_015668360.1	XP_015674373.1	XP_015680511.1	XP_015682671.1
	Reverse BLAST result	Nectin-1	Nectin-1/Nectin-2	Nectin-3	Nectin-4	Necl-1	Necl-2	Necl-3	Necl-4	Nectin-1/Nectin-2
	Hit identified as	Nectin-1	Nectin-2	Nectin-3	Nectin-4	Necl-1	Necl-2	Necl-3	Necl-4	

	Domain organization	Ig/Ig/Ig/TM	Ig/Ig/Ig/TM	Ig/Ig/Ig/TM	Ig/Ig/Ig/TM	Ig/Ig/Ig/TM/b4.1bm	Ig/Ig/Ig/TM/b4.1bm	Ig/Ig/Ig/TM/b4.1bm	Ig/Ig/Ig/TM/b4.1bm	Ig/Ig/Ig/TM
X.laevis	RefSeq protein name	uncharacterized protein LOC494856	Nectin-2	Nectin-3-like	Nectin-4-like	CADM-3	CADM-1-like/homolog	CADM-2-like	CADM-4	Nectin-2
	RefSeq protein id	XP_018080065.1	NP_001184211.1	XP_018101952.1	XP_018089148.1	NP_001080468.1	NP_001090152.1	XP_018104009.1	NP_001087300.1	NP_001184211.1
	Reverse BLAST result	Nectin-1	Nectin-2	Nectin-3	Nectin-4	Necl-1	Necl-2	Necl-3	Necl-4	Nectin-2
	Hit identified as	Nectin-1	Nectin-2	Nectin-3	Nectin-4	Necl-1	Necl-2	Necl-3	Necl-4	
	Domain organization	Ig/Ig/Ig/TM	Ig/Ig/Ig/TM	Ig/Ig/Ig/TM	Ig/Ig/Ig/TM	Ig/Ig/Ig/TM/b4.1bm	Ig/Ig/Ig/TM/b4.1bm	Ig/Ig/Ig/TM/b4.1bm	Ig/Ig/Ig/TM/b4.1bm	Ig/Ig/Ig/TM
L.chalumnae	RefSeq protein name	Nectin-1	Nectin-1-like	Nectin-3	Nectin-4	CADM-3	CADM-1	cell adhesion molecule 2-like	CADM-4	Nectin-1-like
	RefSeq protein id	XP_014350520.1	XP_005999183.1	XP_005993673.1	XP_014349319.1	XP_005995445.1	XP_005988246.1	XP_014353374.1	XP_014345125.1	XP_005999183.1
	Reverse BLAST result	Nectin-1	Nectin-1/Nectin-2	Nectin-3	Nectin-4	Necl-1	Necl-2	Necl-3	Necl-4	Nectin-1/Nectin-2

	Hit identified as	Nectin-1		Nectin-3	Nectin-4	Necl-1	Necl-2	Necl-3	Necl-4	
	Domain organization	Ig/Ig/Ig/TM	Ig/Ig/Ig/TM	Ig/Ig/Ig/TM	Ig/Ig/Ig/TM	Ig/Ig/Ig/TM/b4.1bm	Ig/Ig/Ig/TM/b4.1bm	Ig/Ig/Ig/TM	Ig/Ig/Ig/TM/b4.1bm	Ig/Ig/Ig/TM
D.rerio	RefSeq protein name	Nectin-1	Nectin-2-like	nectin-3-like	Nectin-4	CADM-3	CADM-1	CADM-2a	CADM-4	PVR-like
	RefSeq protein id	NP_001161041.1	XP_021322531.1	NP_001014326.1	XP_001334985.6	NP_001038711.1	XP_005161254.1	XP_005155321.1	NP_001007429.1	XP_021322530.1
	Reverse BLAST result	Nectin-1	Nectin-2/Nectin-1/PVR	Nectin-3	Nectin-4	Necl-1	Necl-2	Necl-3	Necl-4	Nectin-2
	Hit identified as	Nectin-1		Nectin-3	Nectin-4	Necl-1	Necl-2	Necl-3	Necl-4	
	Domain organization	Ig/Ig/Ig/TM	Ig/Ig/Ig/TM	Ig/Ig/Ig/TM	Ig/Ig/Ig/TM	Ig/Ig/Ig/TM/b4.1bm	Ig/Ig/Ig/TM/b4.1bm	Ig/Ig/Ig/TM/b4.1bm	Ig/Ig/Ig/TM/b4.1bm	Ig/Ig/Ig/TM
S.formosus	RefSeq protein name	Nectin-1	Nectin-2-like	nectin-3-like	Nectin-4	CADM-3	CADM-1-like	CADM-2	CADM-4	PVR-like+G80C92:K97
	RefSeq protein id	XP_018620319.1	XP_018587531.1	XP_018614345.1	XP_018593864.1	XP_018613525.1	XP_018610929.1	XP_018619439.1	XP_018587768.1	XP_018587530.1

	Reverse BLAST result	Nectin-1	Nectin-1/Nectin-2/Nectin-3	Nectin-3	Nectin-4	Necl-1	Necl-2	Necl-3	Necl-4	Nectin-1/Nectin-2/Nectin-3
	Hit identified as	Nectin-1		Nectin-3	Nectin-4	Necl-1	Necl-2	Necl-3	Necl-4	
	Domain organization	Ig/Ig/Ig/TM	Ig/Ig/Ig/TM	Ig/Ig/Ig/TM	Ig/Ig/Ig/TM	Ig/Ig/Ig/TM/b4.1bm	Ig/Ig/Ig/TM/b4.1bm	Ig/Ig/Ig/TM/b4.1bm	Ig/Ig/Ig/TM/b4.1bm	Ig/Ig/Ig/TM
C.milli	RefSeq protein name	Nectin-1 [Partial]	Nectin-1	nectin-3-like	Nectin-3-like	CADM-2	CADM-1	CADM-2	CADM-2	Nectin-1
	RefSeq protein id	XP_007910170.1	XP_007910170.1	XP_007891697.1	XP_007891697.1	XP_007901763.1	XP_007894301.1	XP_007901762.1	XP_007901763.1	XP_007910170.1
	Reverse BLAST result	Nectin-1	Nectin-1	Nectin-3	Nectin-3	Necl-1	Necl-2	Necl-3	Necl-1	Nectin-1
	Hit identified as	Nectin-1		Nectin-3		Necl-1	Necl-2	Necl-3		
	Domain organization	Ig/Ig	Ig/Ig	Ig/Ig/Ig/TM	Ig/Ig/Ig/TM	Ig/Ig/Ig/TM/b4.1bm	Ig/Ig/Ig/TM/b4.1bm	Ig/Ig/Ig/TM/b4.1bm	Ig/Ig/Ig/TM/b4.1bm	Ig/Ig
C.intestinalis	RefSeq protein name	PVR	Hemicentin-1	PVR	basement membrane-specific heparan sulfate proteoglycan core protein	CADM-3	CADM-2	CADM-2	CADM-3	Hemicentin-1

	RefSeq protein id	XP_018669328.1	XP_002126696.1	XP_018669328.1	XP_018666843.1	XP_018667030.1	XP_002126544.1	XP_018667030.1	XP_018667030.1	XP_002126696.1
	Reverse BLAST result	Nectin-3/nectin-1	heparan sulfate proteoglycan / hemicentin-1	Nectin-3/nectin-1	basement membrane-specific heparan sulfate proteoglycan core protein	Necl-3	Necl-3	Necl-3	Necl-3	heparan sulfate proteoglycan / hemicentin-1
	Hit identified as							Necl-3		
	Domain organization	Ig*3/dnaA	Ig*5/TM	Ig*3/dnaA	LDLa*3/Ig/LamB/EGF*2/LamB/EGF*4/LamB/EGF*3/Ig*12/LamG/EGF*2/LamG*2	Ig/Ig/Ig/TM	Ig/Ig/Ig/TM	Ig/Ig/Ig/TM	Ig/Ig/Ig/TM	Ig*5/TM
B.floridae	RefSeq protein name	hypothetical protein	hypothetical protein	hypothetical protein	hypothetical protein	hypothetical protein	hypothetical protein	hypothetical protein	hypothetical protein	hypothetical protein
	RefSeq protein id	XP_002603279.1	XP_002599043.1	XP_002601233.1	XP_002601233.1	XP_002587799.1	XP_002598849.1	XP_002599043.1	XP_002599043.1	XP_002598849.1
	Reverse BLAST result	Hemicentin-1	kin of IRRE-like protein 2	Nectin-3	Nectin-3	ran GTPase-activating protein 1	kin of IRRE-like protein 2	Hemicentin-2-like/	Hemicentin-2-like/	kin of IRRE-like protein 2
	Hit identified as									
	Domain organization	Ig*3/TM/SH3/TM/Ig*3/TM/SH3	Ig*3/TM	Ig*3/TM/SH3	Ig*3/TM/SH3	Ig*5/LLR*9	Ig*5/TM	Ig*5/TM	Ig*5/TM	Ig*5/TM

S.kowalevskii	RefSeq protein name	kin of IRRE-like	Obscurin-like	basement membrane-specific heparan sulfate proteoglycan core protein-like	Hemicentin-1-like	Nephrin-like	roundabout-homolog-2-like	Hemicentin-1-like	kin of IRRE-like	Hemicentin-1-like
	RefSeq protein id	NP_001164704.1	XP_002738965.2	XP_006818675.1	XP_002733470.1	XP_006819647.1	XP_006816881.1	XP_006819856.1	NP_001164704.1	XP_002733470.1
	Reverse BLAST result	kin of IRRE-like protein 3	peroxidasin-like	basement membrane-specific heparan sulfate proteoglycan core protein-like	Hemicentin-1	Nephrin	limbic system-associated membrane protein isoform 1 preproprotein [Homo sapiens]	Hemicentin-1	kin of IRRE-like protein 3	Hemicentin-1
	Hit identified as									
	Domain organization	Ig/Ig/Ig/Ig/Ig/Ig/TM	Ig/Ig/Ig/Ig/TM	Ig*6/TM	Ig*12/TM	Ig*9/FN3/TM	Ig*4/FN3	Ig*20/TM	Ig*6/TM	Ig*12/TM
A.planci	RefSeq protein name	CADM-3-like	uncharacterized protein LOC110986899	Nephrin-like	deleted in malignant brain tumors 1 protein-like	uncharacterized protein LOC110978839	Nephrin-like	Nephrin-like	uncharacterized protein LOC110974475	CADM-3-like
	RefSeq protein id	XP_022111393.1	XP_022104883.1	XP_022110423.1	XP_022109510.1	XP_022089818.1	XP_022110423.1	XP_022110423.1	XP_022081834.1	XP_022109091.1
	Reverse BLAST result	kin of irregular chiasm 2	Toll like receptor-1	Nephrin	scavenger receptor cysteine-rich type 1 protein M160	kin of IRRE-like protein 1	Nephrin	Nephrin	cell adhesion molecule 2	kin of IRRE-like protein 3
	Hit identified as									

	Domain organization	Ig/Ig/TM	EGF-like/Ig/Ig/Ig/Ig/TM/TIR	TM/Ig*9/FN3/TM/snRNP or DUF1777	[FU+SRCR]/Ig*4/TM	Ig*3/TM/TM/Ig*3/TM/Ig*3/TM	Ig*9/FN3/TM	Ig*9/FN3/TM	Ig/Ig/Ig/TM	Ig/Ig/TM
S.purpuratus	RefSeq protein name	uncharacterized protein LOC576481	hemicentin-1	Basement membrane-specific heparan sulfate proteoglycan core protein	hemicentin-1	Nephrin	Nephrin	kin of IRRE-like protein 3	Basement membrane-specific heparan sulfate proteoglycan core protein	hemicentin-1
	RefSeq protein id	XP_781880.3	XP_011662955.1	XP_003727633.2	XP_011662955.1	XP_011669820.1	XP_011669820.1	XP_003723913.1	XP_003727633.2	XP_011662955.1
	Reverse BLAST result	vascular cell adhesion protein 1	hemicentin-1	Basement membrane-specific heparan sulfate proteoglycan core protein	hemicentin-1	Nephrin	Nephrin	kin of IRRE-like protein 3	Basement membrane-specific heparan sulfate proteoglycan core protein	hemicentin-1
	Hit identified as									
	Domain organization	Ig	Multiple Ig, EGF and TSP1 domains	Ig*11/LamG*3/EGF*3	Multiple Ig, EGF and TSP1 domains	Ig*9/FN3/TM	Ig*9/FN3/TM	Ig*4/CyoA/PHA02896/TM	Ig*11/LamG*3/EGF*3	Multiple Ig, EGF and TSP1 domains
L.anatina	RefSeq protein name	hemicentin-1	uncharacterized protein LOC106178123	basement membrane-specific heparan sulfate proteoglycan core protein	leucine-rich repeats and immunoglobulin-like domains protein 3	Nephrin	kin of IRRE-like protein 1	CADM-2	kin of IRRE-like protein 1	uncharacterized protein LOC106178123
	RefSeq protein id	XP_013383963.1	XP_013416635.1	XP_023933565.1	XP_013398463.1	XP_013380215.1	XP_013380223.1	XP_013408239.1	XP_013380223.1	XP_013416635.1
	Reverse BLAST result	Hemicentin-1/Hemicentin-2	F11 receptor	basement membrane-specific heparan sulfate proteoglycan core protein	leucine-rich repeats and immunoglobulin-like domains protein 3	Nephrin	kin of IRRE like	opioid-binding protein/cell adhesion molecule	kin of IRRE like	F11 receptor

Species	Row									
	Hit identified as									
	Domain organization	TM/Ig*6/FN3/TM	Ig*3/TM	Multiple LamB/G, Ig and EGF domains	LLR/Ig*5/TM	Ig*9/FN3/TM	Ig*5/TM	Ig/TM/Ig*5	Ig*5/TM	Ig*3/TM
C.gigas	RefSeq protein name	Nectin-1-like	CADM-3-like	kin of IRRE-like protein 1	papilin	protein amalgam	protein amalgam	limbic system-associated membrane protein-like	kin of IRRE-like protein 1	kin of IRRE-like protein 1
	RefSeq protein id	XP_019928344.1	XP_019921398.1	XP_019921374.1	XP_019928300.1	XP_011425709.1	XP_011425709.1	XP_011417286.1	XP_019921374.1	XP_019921374.1
	Reverse BLAST result	Nectin-1	carcinoembryonic antigen-related cell adhesion molecule 1	NEPH1	papilin	Neurotrimin	Neurotrimin	opioid-binding protein/cell adhesion molecule	NEPH1	NEPH1
	Hit identified as									
	Domain organization	Ig/Ig/Ig/TM	Ig/Ig/Ig/TM	Ig*5/TM	Multiple KU, FN3, TSP, LDLa domains/Ig*3/PLAC/TM	Ig/Ig/Ig/TM	Ig/Ig/Ig/TM	Ig/Ig/Ig/TM	Ig*5/TM	Ig*5/TM
H.robusta	RefSeq protein name	-	-	-	hypothetical protein	hypothetical protein	hypothetical protein	hypothetical protein	hypothetical protein	-
	RefSeq protein id				XP_009013731.1	XP_009026168.1	XP_009031547.1	XP_009023684.1	XP_009026168.1	

	Reverse BLAST result				Down syndrome cell adhesion molecule 1	limbic system-associated membrane protein 1	Hemicentin	neurotrimin	limbic system-associated membrane protein 1	
	Hit identified as									
	Domain organization				Ig*8/FN3*4/Ig*2/FN3*2/TM	Ig*3	Ig*2/TSP1*5	Ig*3	Ig*3	
S.mansoni	RefSeq protein name	-	-	axon guidance protein	titin	titin	titin	titin	titin	-
	RefSeq protein id			XP_018652476.1	XP_018655653.1	XP_018655653.1	XP_018655653.1	XP_018655653.1	XP_018655653.1	
	Reverse BLAST result			roundabout holologue 2	hemicentin 1	hemicentin 1	hemicentin 1	hemicentin 1	hemicentin 1	
	Hit identified as									
	Domain organization			Ig*5/FN3/TM	Ig*20/TSP1*5/EGF*7/TM	Ig*20/TSP1*5/EGF*7/TM	Ig*20/TSP1*5/EGF*7/TM	Ig*20/TSP1*5/EGF*7/TM	Ig*20/TSP1*5/EGF*7/TM	
A.mellifera	RefSeq protein name	irregular chiasm C-roughest protein	irregular chiasm C-roughest protein	hemicentin-1	hemicentin-1	irregular chiasm C-roughest protein	nephrin	neurotrimin	hemicentin-1	irregular chiasm C-roughest protein

	RefSeq protein id	XP_006568231.2	XP_006568231.2	XP_003251706.2	XP_003251706.2	XP_006568231.2	XP_006564422.1	XP_006569751.2	XP_006567200.2	XP_006568231.2
	Reverse BLAST result	NEPH-1	NEPH-1	kin of IRRE-like protein 2	kin of IRRE-like protein 2	NEPH-1	Nephrin	Neurotrimin	Hemicentin-2	NEPH-1
	Hit identified as									
	Domain organization	Ig*5/TM	Ig*5/TM	Ig*4/TM	Ig*4/TM	Ig*5/TM	Ig*9/FN3/TM	Ig*3	Ig*7/FN3/TM	Ig*5/TM
B.mori	RefSeq protein name	irregular chiasm C-roughest protein	irregular chiasm C-roughest protein	hemicentin-2	leucine-rich repeats and immunoglobulin-like domains protein 3	irregular chiasm C-roughest protein	lachesin-like	irregular chiasm C-roughest protein	nephrin	irregular chiasm C-roughest protein
	RefSeq protein id	XP_021202791.1	XP_021202788.1	XP_021204268.1	XP_004923208.1	XP_021202791.1	XP_012544014.2	XP_021202791.1	XP_021209075.1	XP_021202791.1
	Reverse BLAST result	NEPH-1	NEPH-1	kin of IRRE-like protein 3	leucine-rich repeats and immunoglobulin-like domains protein 3	NEPH-1	Neurotrimin	NEPH-1		NEPH-1
	Hit identified as									
	Domain organization	Ig*5/TM	Ig*5/TM	Ig*5/TM	LLR*14/Ig*3/TM	Ig*5/TM	Ig*3	Ig*5/TM	Ig*8/FN3/TM	Ig*5/TM

D.melanogaster	RefSeq protein name	Kin of IRRE	Kin of IRRE	Kin of IRRE	lachesin	sticks and stones	sticks and stones	Dpr-interacting protein theta	sticks and stones	Kin of IRRE
	RefSeq protein id	NP_001259196.1	NP_001259196.1	NP_001259196.1	NP_523713.2	NP_788286.1	NP_788286.1	NP_723103.1	NP_788286.1	NP_001259196.1
	Reverse BLAST result	NEPH1	NEPH1	NEPH1	neuronal growth regulator 1	nephrin	nephrin	neurotrimin	nephrin	NEPH1
	Hit identified as									
	Domain organization	Ig*5/TM	Ig*5/TM	Ig*5/TM	Ig*3	Ig*9/FN/TM	Ig*9/FN/TM	Ig*3	Ig*9/FN/TM	Ig*5/TM
A.gambiae	RefSeq protein name	AGAP000217-PA	AGAP000218-PA	AGAP000218-PA	AGAP007928-PA	AGAP001824-PA	AGAP000217-PA	AGAP010742-PA	AGAP010742-PA	AGAP000218-PA
	RefSeq protein id	XP_310917.4	XP_310916.5	XP_310916.5	XP_317553.4	XP_321229.5	XP_310917.4	XP_311455.4	XP_311455.4	XP_310916.5
	Reverse BLAST result	Kin of IRRE-like	Kin of IRRE-like	Kin of IRRE-like	Protein turtle homolog	Hemicentin-1	Kin of IRRE-like	Nephrin	Nephrin	Kin of IRRE-like
	Hit identified as									

	Domain organization	TM/Ig*5/TM	Ig*5/TM	Ig*5/TM	Ig*5/FN3/TM/TM	Ig*5/TM	TM/Ig*5/TM	Ig*9/FN3/TM	Ig*9/FN3/TM	Ig*5/TM
C.elegans	RefSeq protein name	-	Basement membrane proteoglycan	High Incidence of Males	High Incidence of Males	Synaptogenesis protein syg-2	High Incidence of Males	Synaptogenesis protein syg-2	Synaptogenesis protein syg-1	ImmunoGlobulin-like Cell adhesion Molecule family
	RefSeq protein id		NP_001254446.1	NP_509635.2	NP_509635.2	NP_001309467.1	NP_509635.2	NP_001309467.1	NP_508457.3	NP_508166.1
	Reverse BLAST result		basement membrane-specific heparan sulfate proteoglycan core protein	Hemicentin-1	Hemicentin-1	nephrin	Hemicentin-1	nephrin	hemicentin-2	ImmunoGlobulin-like Cell adhesion Molecule family
	Hit identified as									
	Domain organization		Ig/LDLa*3/Ig/EGF/LamB/EGF/LamB/EGF*4/Ig*14/TM	VWA/Ig*47/EGF*2	VWA/Ig*47/EGF*2	Ig*8/FN	VWA/Ig*47/EGF*2	Ig*8/FN	Ig*5/TM	Ig*7/FN3/TM
N.vectensis	RefSeq protein name	-		hypothetical protein	hypothetical protein	hypothetical protein	hypothetical protein	hypothetical protein	hypothetical protein	
	RefSeq protein id			XP_001624042.1	XP_001640242.1	XP_001632489.1	XP_001621716.1	XP_001641619.1	XP_001632489.1	
	Reverse BLAST result			Hemicentin-1	leucine-rich repeats and immunoglobulin-like domains protein 3	basement membrane-specific heparan sulfate proteoglycan core protein	PXDN protein	Hemicentin	basement membrane-specific heparan sulfate proteoglycan core protein	

	Hit identified as									
	Domain organization			Ig*4/FN3/TM	LLR*2/Ig*3/TM	TM/Ig*3	Ig*3/TM	Ig*6	TM/Ig*3	
S.pistillata	RefSeq protein name	basement membrane-specific heparan sulfate proteoglycan core protein	fibroblast growth factor receptor 2-like	hemicentin-2-like	hemicentin-1-like	hemicentin-2-like	hemicentin-2-like	tyrosine-protein phosphatase Lar-like	basement membrane-specific heparan sulfate proteoglycan core protein	fibroblast growth factor receptor 2-like
S.pistillata	RefSeq protein id	XP_022788354.1	XP_022807707.1	XP_022800132.1	XP_022787964.1	XP_022800132.1	XP_022800132.1	XP_022800134.1	XP_022800614.1	XP_022807707.1
S.pistillata	Reverse BLAST result	basement membrane-specific heparan sulfate proteoglycan core protein	Proto-oncogene tyrosine-protein kinase receptor Ret	Hemicentin-1	angiopoietin-related protein 7	Hemicentin-1	Hemicentin-1	receptor-type tyrosine-protein phosphatase delta	basement membrane-specific heparan sulfate proteoglycan core protein	Proto-oncogene tyrosine-protein kinase receptor Ret
S.pistillata	Hit identified as									
S.pistillata	Domain organization	Multiple Ig, LamB, LamG and EGF domains	Ig*2/TM/PTKC	Ig*12	Ig*3/Fibrinogenin	Ig*12	Ig*12	Ig*15/FN3*4/PTP*2	Multiple Ig, LamB, LamG and EGF domains	Ig*2/TM/PTKC
H.vulgaris	RefSeq protein name	hemicentin-2-like	-	hemicentin-2-like	basement membrane-specific heparan sulfate proteoglycan core protein-like	hemicentin-2-like	hemicentin-2-like	basement membrane-specific heparan sulfate proteoglycan core protein-like	hemicentin-2-like	-
H.vulgaris	RefSeq protein id	XP_012556231.1		XP_012556231.1	XP_004211482.2	XP_012556231.1	XP_012556231.1	XP_004211405.2	XP_012556231.1	

	Reverse BLAST result	Hemicentin-2		Hemicentin-2	basement membrane-specific heparan sulfate proteoglycan core protein	Hemicentin-2	Hemicentin-2	basement membrane-specific heparan sulfate proteoglycan core protein	Hemicentin-2	
	Hit identified as									
	Domain organization	Ig*7/FN3*2/TM		Ig*7/FN3*2/TM	LamG/EGF*2/LamG/EGF*2/LamG	Ig*7/FN3*2/TM	Ig*7/FN3*2/TM	LamB/EGF*2/Ig*9	Ig*7/FN3*2/TM	
A.queenslandica	RefSeq protein name	-	Down syndrome cell adhesion molecule-like protein 1 homolog	Down syndrome cell adhesion molecule-like protein 1 homolog	mucin-3A-like	Down syndrome cell adhesion molecule-like protein 1 homolog	mucin-3A-like	mucin-3A-like	mucin-3A-like	Down syndrome cell adhesion molecule-like protein 1 homolog
	RefSeq protein id		XP_019851135.1	XP_019851135.1	XP_019851107.1	XP_019851135.1	XP_019851107.1	XP_019851107.1	XP_019851107.1	XP_019851135.1
	Reverse BLAST result		Hemicentin-1	Hemicentin-1	Hemicentin-1	Hemicentin-1	Hemicentin-1	Hemicentin-1	Hemicentin-1	Hemicentin-1
	Hit identified as									
	Domain organization		Ig*9/FN3*2/TM	Ig*9/FN3*2/TM	Ig*13/FN*6/TM	Ig*9/FN3*2/TM	Ig*13/FN*6/TM	Ig*13/FN*6/TM	Ig*13/FN*6/TM	Ig*9/FN3*2/TM

Key

Protein name	As annotated in RefSeq
Protein id	As per Refseq
	Represents that the protein is missing in this organism
Red text	Might be some nectin ancestor
Green text	Not paralogue of human nectin, but a nectin

Domains

b4.1 bm	Band 4.1 binding motif
CyoA	Heme/copper-type cytochrome/quinol oxidase, subunit 2
EGF	Epidermal growth factor like domain'
FN3	fibronectin type 3 domain
Ig	Immunoglobulin fold
LamB	Laminin B domain
LamG	Laminin G domain
LDLa	Low-density lipoprotein receptor domain class A
PHA02896	A-type inclusion like protein
PTKC	Protein tyrosine kinase C domain
PTP	Protein tyrosine phosphatase domain
SRCR	scavenger receptor cysteine-rich
TM	Transmembrane region
TSP1	Thrombospondin type 1 repeats
VWA	von Willebrand factor (vWF) type A domain
